# Rising competition among North Sea mammalian top predators: a multi-method perspective on trophic ecology

**DOI:** 10.1038/s41598-026-53094-2

**Published:** 2026-05-14

**Authors:** Eileen Heße, Joy Ometere Boyi, Krishna Das, Kristina Lehnert, Mathilde Piette, Marianna Pinzone, Ursula Siebert, Anita Gilles

**Affiliations:** 1 Institute for Terrestrial and Aquatic Wildlife Research, University of Veterinary Medicine Hannover, Foundation, Büsum, Germany; 2 Wageningen Marine Research, Wageningen University & Research, Den Helder, The Netherlands; 3 Freshwater and Oceanic Sciences Unit of Research, University of Liege, Liege, Belgium; 4 Norwegian Polar Institute, Fram Centre, Tromsø, Norway; 5 Department of Ecoscience, Aarhus University, 8000 Aarhus, Denmark; 6 Present Address: Wageningen Marine Research, Ankerpark 27, 1781 AG Den Helder, The Netherlands

**Keywords:** Niche overlap, Feeding ecology, *Phoca vitulina*, *Halichoerus grypus*, *Phocoena phocoena*, Bayesian modelling, Ecology, Ecology, Ocean sciences, Zoology

## Abstract

Top predators are crucial in shaping ecosystem dynamics by regulating key processes such as prey populations, energy transfer, and community structure, particularly in systems where multiple species compete for the same resources. Understanding their trophic niches and interactions is essential for effective conservation. In the southern North Sea, harbor seals, gray seals, and harbor porpoises are top predators with overlapping prey. This multi-method study examined resource partitioning using complementary stomach content analysis, metabarcoding, and carbon, nitrogen, and sulfur stable isotope analysis to refine habitat use, incorporating sulfur isotopes, for the first time in marine mammals of the southern North Sea. Gastrointestinal data from stranded harbor seals (n = 223), gray seals (n = 87), and harbor porpoises (n = 218), alongside 283 scat samples from wild seals, collected between 2014 and 2021, were analyzed. Harbor seal and gray seal showed high prey similarity (Jaccard index 0.71), while porpoises exhibited lower similarity with both seal species (Jaccard index 0.46 and 0.45). Interactions with prey guilds were strongest for demersal roundfish, flatfish, gobies, and sandeels. Bayesian isotope mixing models revealed consistent trophic differences among the three top predators, with seals occupying higher trophic positions than porpoises and showing minimal dietary change over time. Isotopic niche metrics indicated increasing overlap between porpoises and gray seals, particularly in δ^34^S/δ^15^N space, suggesting growing trophic similarity, while harbor seals showed a contraction in niche area. Also, stomach content data revealed that high-energy prey in porpoises declined as gray seal abundance increased. These results highlight trophic niche overlap and substantial interspecific interactions, potentially leading to competition under limited resources.

**Supplementary Information:**

The online version contains supplementary material available at 10.1038/s41598-026-53094-2.

## Introduction

Top predators play a central role in ecosystem dynamics, with trophic interactions regulating key processes such as prey populations, energy transfer, and community structure^1–3^. In ecosystems with several predator species with similar functional traits, competition for resources and trophic partitioning can influence the roles that different species play^4,5^. Identifying trophic niches of top predators and linkages between them is crucial to avoid mismanagement of already limited conservation funds^6^.

Species coexistence relies on resource partitioning^7^, but distinguishing between competition and coexistence has proven challenging^8^. Here, we define interspecific competition as individuals from different species competing for the same resources^9^. To be successful, niche separation needs to occur in some or all niche dimensions such as foraging in spatially distinct areas or foraging at different times^10,11^. Thus, if resources are sufficiently abundant and dietary plasticity is present, species sharing the same trophic level can reduce the likelihood of interspecific competition by segregating their niches^12^. However, direct identification of competition in the wild is often challenging, especially when studying highly mobile and elusive species such as marine mammals^13^. It requires detailed knowledge of predator–prey interactions and available resources in the ecosystem. In the North Sea, marine mammal abundances are well studied^14–16^ while data on prey abundance is almost exclusively available for species of commercial interest^17^.

Being an extremely productive and facilitating area with high energy densities of prey species, the North Sea constitutes a favorable habitat for higher trophic species^18^. Yet, the North Sea is heavily influenced by anthropogenic activities and climate change^19^, potentially altering prey availability and ultimately intensifying competition. Gray seals (*Halichoerus grypus*), harbor seals (*Phoca vitulina*), and harbor porpoises (*Phocoena phocoena*) overlap in spatial distribution and prey resources in the southern North Sea^20,21^. Previous studies have only focused on interspecific competition between the two sympatric pinniped species^13,22^.

Both seal species and the harbor porpoise have high energy demands with fish being their common primary food source in the southern North Sea^20,23,24^. Harbor seals and gray seals mainly feed on flatfish, demersal roundfish, gadoids, and sandeels in shallow, soft-sediment habitats^23,25,26^. Harbor porpoises exhibit a broad diet including pelagic and benthic prey like flatfish, sandeels, gadoids, gobies, and clupeids^24,27^.

The objective of this study was to assess resource partitioning among harbor seals, gray seals, and harbor porpoises by analyzing their dietary profile, considering several dimensions of their trophic niches. Given that these marine mammals appear to feed largely on the same prey guilds, we hypothesized that isotopic niches of the three top predator species overlap. To test this hypothesis, we applied a complementary approach including stomach content analysis, metabarcoding and stable isotope analysis to examine dietary composition and isotopic niche overlap, with this study being the first to incorporate sulfur isotopes to refine foraging habitat use of the three top predators.

## Materials and methods

### Sample collection

#### Samples from stranded marine mammals

**Fig. 1:**
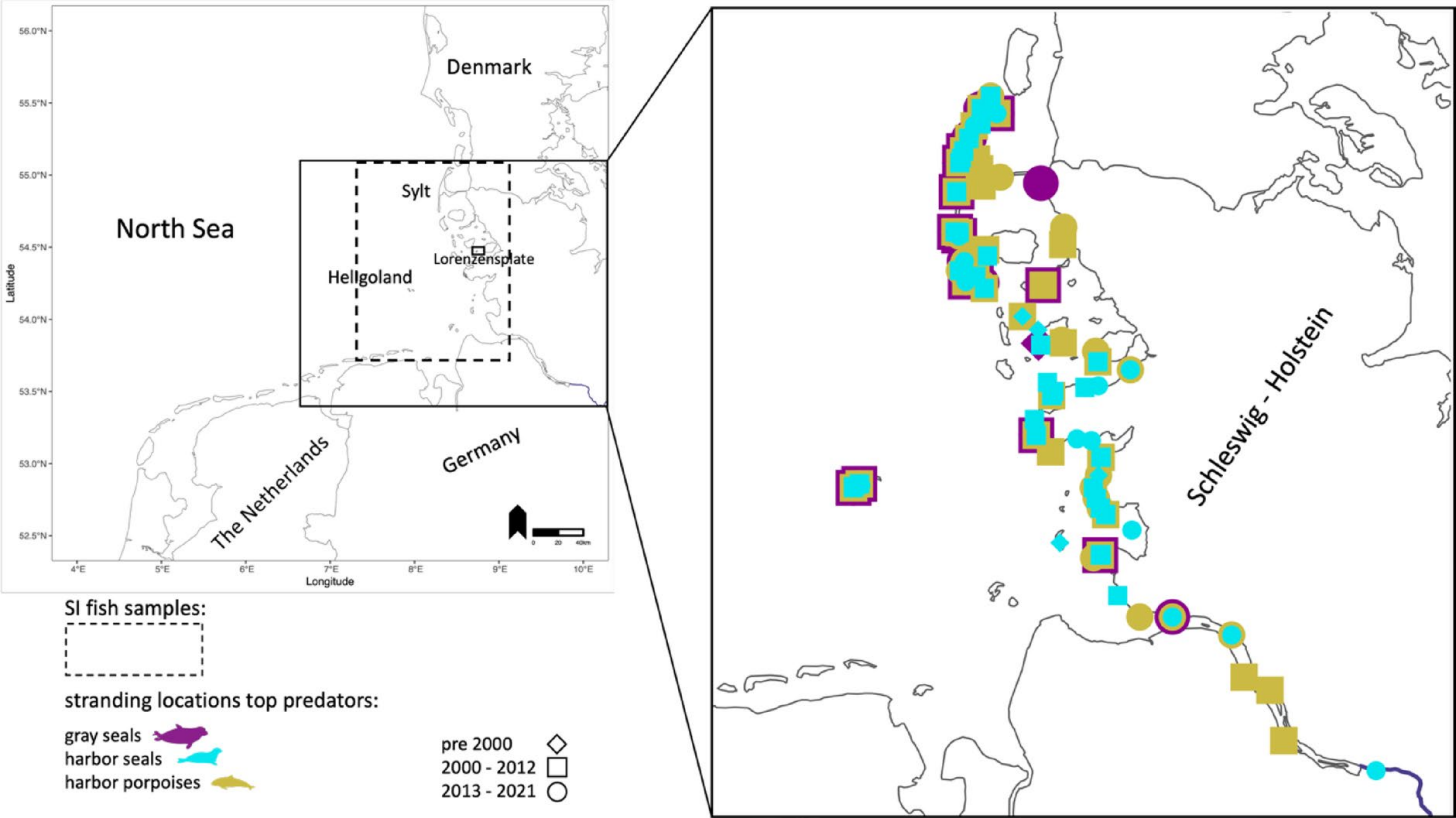
Overlapping stranding locations of harbor porpoises (green, n = 218), harbor seals (turquoise, n = 223) and gray seals (purple, n = 87) included in this study. Samples were divided into three different sampling periods: pre 2000 (diamond), 2000–2012 (square) and 2013–2021 (circle). The study area where fish (prey) was sampled for stable isotopes is indicated by the dashed rectangle^31^. Scat samples for metabarcoding were collected on Heligoland and the Lorenzensplate.

Since the 1990s, stranded harbor porpoises, gray seals and harbor seals along the coastline of Schleswig–Holstein (Germany) have been necropsied to determine their biological parameters, health status, and/or cause of death as part of a long-term health monitoring program^28,29^. Organs, including muscle, intestine, and stomach, are assessed during necropsies, and samples or complete organs stored at − 20 °C until further analysis^29,30^. Carcasses included in this study cover 218 stranded harbor porpoises, 223 harbor seals (96 stranded, 88 mercy-killed and 39 of unknown origin) and 87 gray seals (67 stranded and 20 mercy-killed), collected between 1994 and 2021 along the North Sea coast of Schleswig–Holstein (Fig. 1, Table 1, Supplementary Table S1). Mercy-killed animals were humanely euthanized by designated and trained seal hunters of Schleswig–Holstein using firearms, following a medical examination^28^. All samples were obtained post-mortem.

**Table 1:** Mean total length (LT) and weight (W) (± s.d.) of gray seal (n = 87), harbor seal (n = 223) and harbor porpoise (n = 218) individuals included in this study.

Species	Age class	Sex	N_sex_	LT(cm)	N_L_	W(kg)	N_W_
Gray seal *Halichoerus grypus*	Adult	♂	10	219 ± 19.6	6	105 ± 41	5
		♀	6	196 ± 11.6	6	111 ± 29.2	6
	Juvenile	♂	31	127 ± 12.9	24	32.8 ± 13.2	19
		♀	15	124 ± 8.83	12	31.4 ± 12.3	8
	Pup	♂	13	102 ± 7.56	11	14 ± 5.18	12
		♀	11	99.9 ± 6.43	5	15.2 ± 8.8	5
Harbor seal *Phoca vitulina*	Adult	♂	22	166 ± 10.9	16	71 ± 21.3	18
		♀	54	161 ± 11.4	46	67.7 ± 18.4	51
	Juvenile	♂	55	109 ± 9.14	51	18.6 ± 5.43	50
		♀	49	107 ± 9.32	44	18.5 ± 6.75	43
	Pup	♂	22	91.3 ± 3.91	11	9.25 ± 2.36	15
		♀	18	86.1 ± 5.56	12	8.06 ± 1.67	13
Harbor porpoise *Phocoena phocoena*	Adult	♂	41	137 ± 5.84	30	42 ± 7.22	16
		♀	41	147 ±—8.78	34	51.5 ± 11.9	19
	Juvenile	♂	52	111 ± 9.86	46	20.2 ± 7.26	32
		♀	45	111 ± 8.77	45	20.3 ± 5.99	28
	Neonate	♂	14	84.6 ± 7.46	14	9.67 ± 3.10	11
		♀	19	83 ± 9.67	19	9.95 ± 3.34	11

N_sex_ is the number of animals available per sex (not all individuals could be sexed). N_L_ is the number of animals with available length measurements and N_W_ the number of animals weighed. Sex identification was missing for one gray seal, three harbor seals and six harbor porpoises. LT(cm) is the lotal length in cm and W(kg) is the total weight in kg.

For harbor porpoises, classification into three age categories (neonate, juvenile, and adult) was based on measured body lengths, status of reproduction organs or age determination by evaluating annual dental growth layers^29^. Both pinniped species were categorized as either pup, juvenile or adult depending on their length, sex and date of stranding, thereby assessing the reproductive season relative to the time of the year (Supplementary Table S2). For both grey seals and harbor seals, although less pronounced in the latter, males are larger than females. As such age class categorization was conducted considering the sexual dimorphism, following^32,33^. Only data on juvenile and adult top predators were included in this study to avoid biases introduced through nursing^34,35^. Length categories were adapted from^36^; however, we did not further differentiate between subadults and adults as data on sexual maturity were often missing.

#### Scat samples

A total of 153 scat samples from wild harbor seals were gathered from haul-out sites between 2014 and 2019 during spring (March—May) and autumn (September—October) as part of ongoing seal health monitoring on the Lorenzensplate, a sandbank in the North Sea^37^ (Fig. 1). In addition, scats from 131 gray seal individuals were collected on the offshore island Heligoland in the North Sea during various periods: in 2010–2011 (May, July, and, September), 2017 (March), and 2020–2021 (November–April) (Fig. 1). Furthermore, two gray seal scats were obtained from the sandbank (Lorenzensplate) in April 2015. During sample collection, it was assumed that each scat originated from a distinct individual. Harbor seal scats were assumed to be no older than approximately 6 h at the time of collection, based on the 6 h tidal cycle at the sandbank, where haul-out sites are fully submerged during high tide. In contrast, gray seal scat freshness was assessed in the field based on a subjective classification (fresh, medium, old), as beaches on Heligoland are not fully inundated during high tide and scats may persist longer. Freshness was not explicitly controlled for in downstream analyses. However, all samples, irrespective of assigned freshness category, yielded successful amplification, suggesting that potential bias due to DNA degradation is likely limited (see also^26^). All seal scat samples were promptly stored at − 20 °C until DNA isolation. A subset of these scats was previously analyzed by^26^.

Gray and harbor seal scats, indistinguishable in the field, were genetically assigned to species based on a 220 bp fragment of the mitochondrial 16S gene amplified using primers PINNL and PINNR^38^. PCR products were visualized on agarose gels, purified, and Sanger sequenced. Sequences were aligned to GenBank references in Clustal Omega (Geneious Prime v2022.0.2) for species identification. For detailed procedures, see^26^.

### Stable isotope analysis

Stable isotope ratios of carbon (δ^13^C), nitrogen (δ^15^N), and sulfur (δ^34^S) were measured on 300 muscle samples of deceased harbor porpoises, gray seals and harbor seals, following established protocols described in^27^, based on^22,39^. Briefly, ~ 200 mg of freeze-dried, homogenized tissue was weighed into tin capsules (with tungsten (W) added for combustion optimization) and analyzed with an IsoPrime PrecisION mass spectrometer coupled to an N–C–S elemental analyzer (Vario MICRO cube). Isotope ratios were expressed in δ notation relative to international standards (Vienna Peedee Belemnite (VPDB) for δ^13^C, Atmospheric Air for δ^15^N, and Vienna-Canyon Diablo Troilite (VCDT) for δ^34^S), with IAEA-certified reference materials (IAEA-C6, IAEA-N2, IAEA-S2). Sulfanilic acid served as primary and secondary standard. Analytical precision for predator samples was 0.2‰ for δ^13^C, δ^15^N, and δ^34^S. Lipid normalization for δ^13^C was performed when the C:N ratio exceeded 3.8 using the correction of^40^, as adapted by^41^; however, all predators had C:N ratios between 3.1 and 3.8. Hence, no lipid correction was required.

Mean isotopic values of δ^13^C were non-normally distributed across all three predator species. In contrast, δ^15^N and δ^34^S were normally distributed in gray seals but showed non-normal distributions in harbor seals and harbor porpoises. Accordingly, subsequent analyses were conducted using non-parametric tests and Bayesian inference.

Fish prey samples were collected within German waters of the North Sea. After collection, fat extraction was performed using a Soxtherm system (type SE406, C. Gerhardt GmbH), and measured on an EA-IRMS (FlashEA 1112, Thermo Fisher Scientific, Bremen, Germany). Analytical precision for prey samples was < 0.15‰ (one standard deviation) for δ^13^C and δ^15^N. A subsample of these data has previously been analyzed in^27^.

All data were analyzed within the R environment (version 4.3.2,^42^). Stable isotope values (δ^13^C, δ^15^N, δ^34^S) were first compared using Kruskal-Wallace tests (Supplementary Table S6) and then jointly modelled implementing a multivariate Bayesian model using the package brms in R (version 2.23.0^43^), which interfaces with Stan^44^. The three isotopes were modelled with Gaussian error distributions, allowing estimation of residual correlations among response variables. Species, sex, age class, and season were included as fixed effects in all models. Time frame was included as a random intercept to account for temporal structure in the data. The model was specified as:

$$\delta^{{{13}}} {\mathrm{C}},\;\delta^{{{15}}} {\mathrm{N}},\;\delta^{{{34}}} {\mathrm{S}}\sim {\mathrm{species}} + {\mathrm{sex}} + {\mathrm{age}} + {\mathrm{season}} + ({1}|{\text{time period}})$$

Models were fitted using four Markov chain Monte Carlo (MCMC) chains with 4000 iterations each, including 2000 warm-up iterations, resulting in 8000 posterior samples. To improve sampling efficiency and avoid divergent transitions, the target acceptance rate was set to 0.995 and the maximum tree depth to 15. Model convergence was assessed using the potential scale reduction factor (R̂) and effective sample size. Model fit was evaluated using posterior predictive checks.

All δ^13^C values were corrected for the oceanic Suess effect^45^ to standardize across sampling years, using the SuessR package in R (version 0.1.5^46^). Data were adjusted to the year 2020, being the average year of collected fish prey stable isotope data.

#### Trophic position and source contribution

Trophic position was calculated using the trps package in R (v 0.1.0;^47^). Bayesian models were implemented in Stan, via the brms interface. The underlying equations for trophic position estimation follow established isotope-based models as described by^48–50^. We used a two-source mixing model with α to estimate the contribution of benthic and pelagic sources, following the parameterization described in^47^, with one exception: the trophic enrichment factor (TEF) for nitrogen was adjusted to account for differences between seals and porpoises. For porpoises, we used the TEF reported by^51^ for fin whale (*Balaenoptera physalus*) muscle tissue, consistent with the approach taken by^27^. For seals, we applied the TEF from^52^ based on captive seal muscle tissue. Baselines were selected in line with^53^: Laver spire shell (*Peringia ulvae*) as the benthic baseline and blue mussel (*Mytilus edulis*) as the pelagic baseline. Mean δ^13^C and δ^15^N values for these baseline species were taken from^53,54^. *M. edulis* was used as a pelagic baseline as it is widely applied in stable isotope studies in the Wadden Sea and southern North Sea as a proxy for suspended particulate organic matter, reflecting integration of water column-derived material. Individuals were collected from deep channel buoys just below the surface^53^; although some source mixing cannot be excluded in this well-mixed system, this approach ensures comparability with previous regional studies (see e.g.,^53,55^).

In addition to conventional summary statistics, Bayesian probability of direction was used as an intuitive measure of effect certainty, complementing effect sizes and credible intervals by quantifying the proportion of posterior samples supporting a consistent effect direction.

#### Isotopic niche overlap using SIBER

Bayesian inference was used to compare isotopic niche overlap among the three predator species across two consecutive time periods (2000–2012 (n = 69) and 2013–2021 (n = 232); Supplementary Table S3) using the SIBER package in R (v2.1.9;^56^). Niche overlap was assessed pairwise (δ^13^C–δ^15^N, δ^13^C–δ^34^S, δ^34^S–δ^15^N) to retain ecological interpretability of distinct niche axes (carbon source, trophic position, and benthic–pelagic coupling), rather than combining all isotopes into a single multivariate metric. Standard ellipse areas (SEA) were calculated as proxies for trophic niche width, with corrected ellipse areas (SEAc) applied to account for differences in sample size^57–59^. 40% ellipses were generated to compare the most representative individuals of a population with the majority of the population (95% ellipses).

#### Potential overlap with prey isotopic niches

Potential overlap with prey isotopic niches (δ^13^C and δ^15^N) was assessed following the method of^13^, which estimates species-level ellipses from posterior distributions accounting for covariance among isotopic values. Isotopic values for simulated individuals were drawn from posterior distributions in 1,000 iterations to quantify overlap uncertainty. Intra-specific niche comparisons between adults and juveniles were performed for individuals sampled between 2013 and 2021, corresponding to the period with available prey data (Supplementary Table S4).

### Stomach content analysis

Stomach contents were available from 24 gray seals, 61 harbor seals, and 103 harbor porpoises and were analyzed following^60,61^. Stomachs were frozen at –20 °C or processed fresh. Intact prey remains were measured and identified directly, while residual contents were rinsed to isolate hard parts such as otoliths and cephalopod beaks, which were identified under a microscope and used to reconstruct prey biomass^62,63^. Prey remains were foremost identified according to^62–64^ and an internal reference collection at the Institute of Terrestrial and Aquatic Wildlife (ITAW) of the University of Veterinary Medicine Hannover, Foundation. Prey remains were photographed and measured using an OLYMPUS UC90 camera and OLYMPUS cellSens software (version 3.2.1; https://evidentscientific.com/en/downloads). Otolith wear was taken into account and subsequently corrected for using correction factors outlined in^24,63^.

For each prey species, frequency of occurrence (FO), percentage frequency of occurrence (%FO), consumed biomass, percentage consumed biomass, and estimated energy contribution (%kJ) were calculated. Dietary importance was assessed using the Index of Relative Importance (IRI = %FO × (%N + %M)), where %FO represents the proportion of stomachs containing a given prey relative to the total number of stomachs, %N is the numerical percentage of that prey relative to the total number of prey items, and %M is the percentage contribution to the total reconstructed prey biomass^65^. Prey guild classification followed^24^, combining ecological and taxonomic criteria.

#### Energy-rich prey in the diet of harbor porpoise

Generalized additive models (GAMs) were used to assess factors influencing the biomass of energy-rich prey (≥ 5 kJ g⁻^1^ wet weight;^24^) in the diet of harbor porpoises stranded between 1998 and 2021. Models were constructed with the mgcv package in R (v1.9–1;^66^). Explanatory variables included sex, age class, gray seal abundance, and month. Annual gray seal abundances for the German North Sea (Lower Saxony, Hamburg, Schleswig–Holstein and Heligoland) were derived from molt counts^67^. Prior to 2006, no internationally coordinated abundance data were available and therefore the abundance was set to 0 for those years. While acknowledging that no dedicated gray seal abundance surveys took place in the Wadden Sea prior to 2006 but individual gray seals already have been present in parts of the Wadden Sea, 0 simply represents low numbers of permanent individuals in the area^34^.

Smooth terms were applied to all variables: sex and age as random effects (bs = “re”), gray seal abundance as a cubic spline basis (bs = “cs”), and month as a cyclic cubic spline (bs = “cc”) to account for seasonality. The smoothness parameter k was adjusted for gray seal abundance and month to allow flexible fitting. Year was excluded due to strong correlation with gray seal abundance (Pearson r = 0.95) and missing data between 2006 and 2011. A quasi-Poisson distribution was used to account for overdispersion.

### Metabarcoding

Metabarcoding was used to only target fish prey species, reflecting the predominant component of the diet, while recognizing that these predators also feed on a broader range of taxa, including crustaceans and cephalopods. These were not captured here since this would require additional primer sets that were not included in this analysis.

A universal 16S rRNA primer, suitable for both marine and freshwater fish prey species, was applied on stomach and/or intestinal samples from 50 harbor porpoises and on scats from 153 harbor seals and 131 gray seals. Details of the metabarcoding approach and primer design can be found in^26^. Briefly, DNA extraction from 200 to 250 mg of digesta was conducted using the QIAamp Fast DNA Stool Mini Kit (Qiagen, Hilden, Germany). Negative controls (blanks) were included in each extraction to monitor for contamination, while vouchered fish species (common carp (*Cyprinus carpio*), rudd (*Scardinus erythropthalmus*) and common roach (*Rutilus rutilus*)) were used for validation and positive controls. Subsequently, DNA was subsampled into two 50 µL volumes to preserve DNA integrity and avoid repeated freeze–thaw cycles, then stored at − 20 °C.

A two-step enrichment PCR strategy was employed to amplify trace amounts of prey DNA efficiently. The first step PCR amplified the target region using locus-specific primers, whereas the second step PCR utilized primers containing both the locus-specific sequence and a universal 5′ tail as specified in the Illumina Nextera library protocol.

The second step PCR enriched the target region using products from the first PCR as templates.

In the third step PCR, unique indices (barcodes) and Illumina adapters were attached to all second step PCR products, followed by further amplification. Paired-end sequencing was performed using an Illumina MiSeq sequencer with a MiSeq Reagent Kit V2 (500 cycles) (2 X 250 bp) (Illumina) at Microsynth Next Generation Facilities, Switzerland.

Following quality control, Operational Taxonomic Units (OTUs) were assigned. OTU sequences were matched against a manually curated reference database derived from NCBI accessions. OTUs were classified at species level using a confidence threshold of > 0.9.

Metabarcoding data were analyzed as percentage frequency of occurrence (%FO). Dietary overlap between the three predators was calculated with presence/absence based on the Jaccard similarity index using the jaccard package (version 0.1.0;^68^).

## Results

### Stable isotope analysis

#### Effects of species and covariates on stable isotope values

Mean and standard deviation (s.d.) for all isotopes per species and sampling period are listed in Table 2 (values per predator’s sex and age class are listed in Supplementary Table S3).

**Table 2:** Mean isotopic values and standard deviation (s.d.) of carbon (δ^13^C), nitrogen (δ^15^N) and sulfur (δ^34^S) isotopes for gray seals, harbor seals and harbor porpoises included in this study.

		2000–2012		2013–2021	
Isotope	Species	mean	n	mean	n
δ^13^C	Gray seal	− 17.8 ± 1.0	12	− 17.7 ± 1.2	43
δ^13^C	Harbor seal	− 16.8 ± 1.8	39	− 16.8 ± 0.9	94
δ^13^C	Harbor porpoise	− 18.4 ± 1.2	17	− 17.9 ± 0.8	95
δ^15^N	Gray seal	18.3 ± 1.6	12	18.9 ± 1.6	43
δ^15^N	Harbor seal	19.2 ± 1.6	39	18.8 ± 1.0	94
δ^15^N	Harbor porpoise	16.7 ± 1.6	17	17.4 ± 1.3	95
δ^34^S	Gray seal	16.9 ± 2.2	12	19.9 ± 2.6	43
δ^34^S	Harbor seal	15.3 ± 1.9	39	18.0 ± 1.1	94
δ^34^S	Harbor porpoise	14.7 ± 2.5	17	19.1 ± 1.4	95

The multivariate Bayesian model identified consistent species differences across all isotopes, whereas effects of age, sex, and season were limited (Supplementary Table S5). Harbor porpoises exhibited lower δ^15^N values than gray seals (mean difference =  − 1.41, 95% CI − 1.87 to − 0.93), while both harbor porpoises and harbor seals showed lower δ^34^S values relative to gray seals (− 1.00, − 1.61 to − 0.39; and − 1.72, − 2.30 to − 1.14, respectively). Differences in δ^13^C among species were less pronounced. Across species, only small effects of covariates were detected, including higher δ^13^C values in adults and lower δ^15^N values in males and in spring (Supplementary Table S5). These statistical differences in bulk isotope ratios were consistent with the variability observed in trophic position and resource use, as described below.

#### Trophic position and source contribution

All p-values reported are Bayesian probabilities of direction, summarizing the proportion of posterior samples supporting a directional effect. These were calculated directly from posterior draws. Bayesian isotope mixing models revealed distinct trophic differences among the three marine mammal species across two periods (2000–2012 and 2013–2021). Harbor porpoises consistently fed at lower trophic positions (TP) than both seal species, with a moderate TP increase from 4.4 to 4.7 and reduced pelagic reliance (α: 0.56 to 0.71; Supplementary Fig. 1). However, this shift showed limited evidence for change (TP overlap = 0.64; p = 0.75). Harbor seals maintained high TPs (5.8 to 5.6) and strongly benthic diets (α: 0.87 to 0.89), with little change over time (overlap = 0.83; p = 0.62; Supplementary Fig. 1). Gray seals were similarly stable (TP: 5.4 to 5.7; α: 0.76 to 0.77; overlap = 0.74; p = 0.68; Supplementary Fig. 1). Trophic differences between gray and harbor seals were small and declined over time (Supplementary Fig. S1).

#### Isotopic niche overlap top predators

The isotopic niche metrics showed clear temporal and species-specific differences. For δ^13^C/δ^15^N, harbor porpoises and gray seals exhibited an increase in total area (TA) over time (18.2 to 24.9 and 10.0 to 22.4, respectively), while harbor seals decreased (36.7 to 22.6) (Supplementary Table S7). Corresponding SEAc declined for porpoises and harbor seals but increased for gray seals. In δ^34^S/δ^15^N space, TA and SEAc increased markedly in gray seals (TA: 16.2 to 53.0; SEAc: 9.8 to 13.3) and modestly in porpoises, but decreased in harbor seals. For δ^13^C/δ^34^S, porpoises and gray seals expanded their niches over time, whereas harbor seals showed a reduction in both TA and SEAc (Supplementary Table S7).

**Fig. 2:**
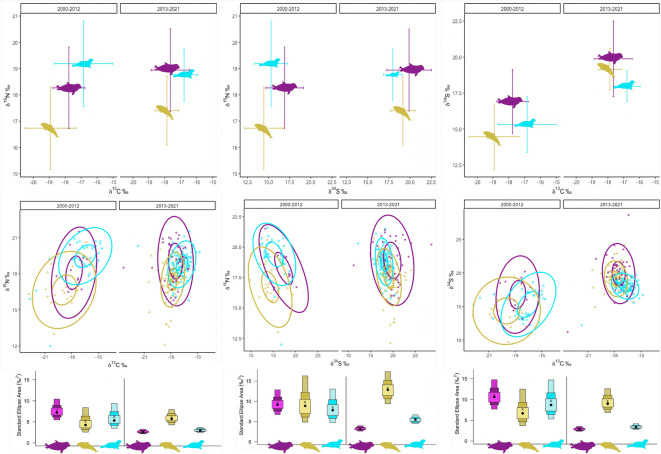
The top row shows mean isotopic values and standard deviations for gray seal (purple), harbor seal (turquoise) and harbor porpoise (green) in two different sampling periods (2000–2012 and 2013–2021) for combinations of δ^13^C/δ^15^N (left), δ^15^N/δ^34^S (middle) and δ^13^C/δ^34^S (right). The middle row illustrates 40% and 95% ellipses for all three predators per sampling period and per isotope combination. The bottom row displays the standard ellipse area for all three predators per sampling period and per isotope combination (black circle is the mode and x is the maximum likelihood (ML) estimated SEA_c_).

Niche overlap varied considerably among species and isotopic dimensions, with patterns indicating increased isotopic niche similarity between harbor porpoises and gray seals over time, particularly in δ^34^S/δ^15^N space, while overlap with harbor seals remained stable or decreased (Fig. 2, Supplementary Table S8). In δ^13^C/δ^15^N, 40% ellipse overlaps were low to moderate, with harbor porpoise—harbor seal overlap declining slightly over time and porpoise—gray seal overlap increasing to ~ 49% of the porpoise ellipse. 95% overlaps in this space remained high, often exceeding 50% and reaching 100% between harbor seals and gray seals. In δ^34^S/δ^15^N, negligible 40% overlaps in the earlier period shifted to higher values in 2013–2021, especially between porpoises and gray seals (~ 45%) and harbor seals and gray seals (> 80%). For δ^13^C/δ^34^S, overlaps declined between porpoises and harbor seals but remained substantial with gray seals, including complete inclusion of the porpoise niche within the gray seal niche in the later period (Fig. 2, Supplementary Table S8).

#### Potential overlap with prey species

**Fig. 3:**
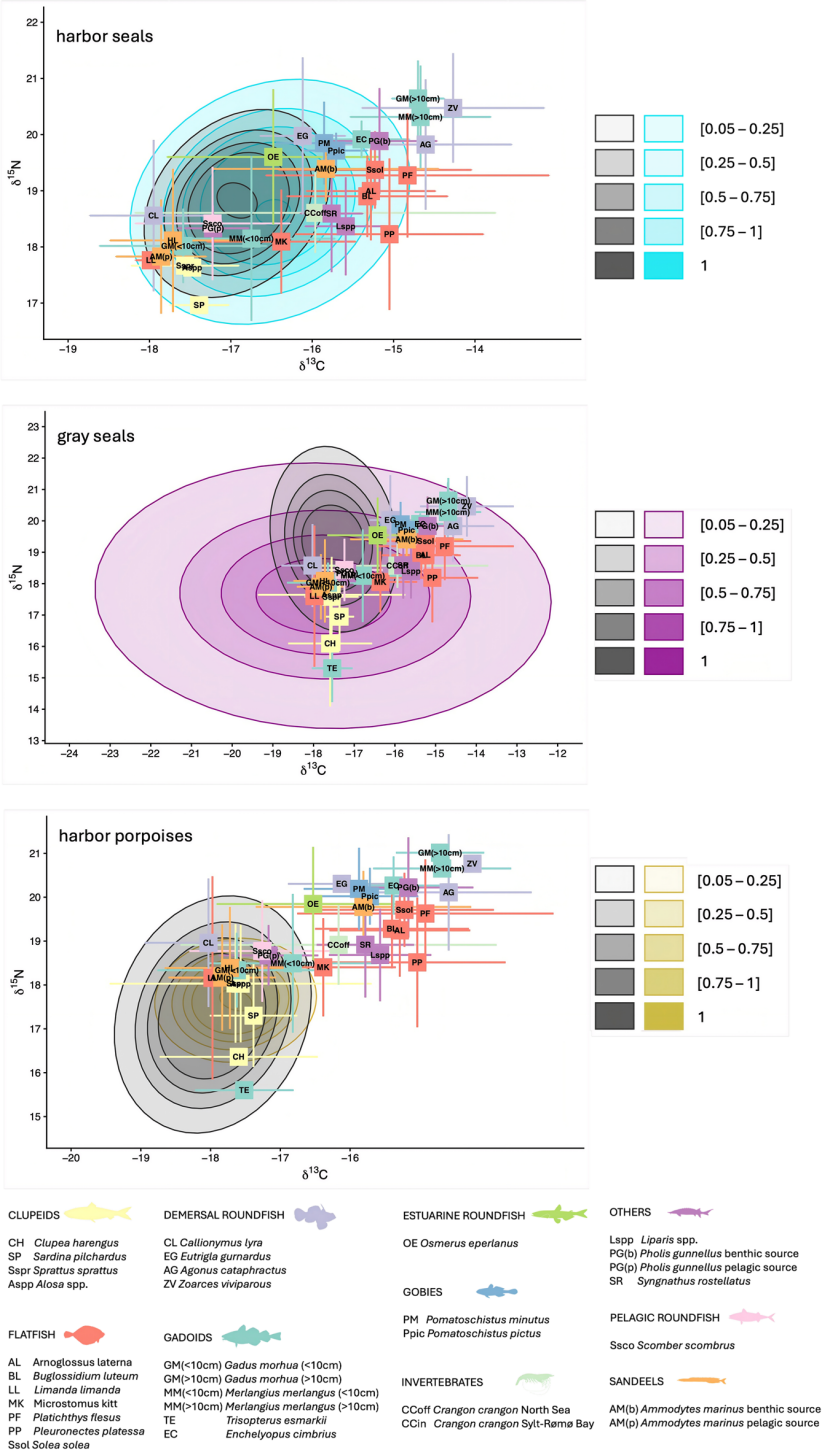
Comparison of isotopic niches of adult and juvenile harbor seals (top), adult and juvenile gray seals (middle) and adult and juvenile harbor porpoises (bottom) with isotopic values of potential prey in the southern North Sea. Gray ellipses represent juveniles and colored ellipses adults (color-coding is alike with Fig. 2). Ellipses show the probability of belonging to the isotopic niche. Prey species are color-coded according to the prey guild they belong to. Invertebrate prey was categorized as offshore (off) and inshore (in). Gray seal and harbor seal trophic enrichment factors (TEFs) are based on^52^ and harbor porpoise TEFs are based on^63^ (muscle).

Potential overlap with prey species could be assessed for 94 harbor seals, 43 gray seals, and 97 harbor porpoises. Adult gray seals and harbor seals had broader isotopic niches compared to juveniles, whereas the pattern was reversed in harbor porpoises (Fig. 3). All available prey species (n = 33) lay within the isotopic niche of gray seals. Mean isotopic values of larger gadoids (> 10 cm; whiting (*Merlangius merlangus*) and Atlantic cod (*Gadus morhua*)) and demersal roundfish (viviparous blenny (*Zoarces viviparous*) and hooknose (*Agonus cataphractus*)) were outside the niche of harbor seals. Adult and juvenile harbor porpoises had niches much narrower than those of the two pinniped species, resulting in less prey species (n = 13) within their niches. The probability for intra-specific overlap in juvenile and adult harbor porpoises was highest for sandeels, clupeids and small Atlantic cod (Supplementary Fig. S2). Juvenile and adult gray seals had the highest probability of intra-specific overlap for butterfish (*Pholis gunnellus*), dragonet (*Calionymus lyra*) and Atlantic mackerel (*Scomber scombrus*). Harbor seals also had the highest probability of intra-specific overlap for butterfish and Atlantic mackerel and additionally for small whiting and smelt (*Osmerus eperlanus*). Overall intra-specific overlap for adults and juveniles was less likely in gray seals followed by harbor seals and with the highest probability in harbor porpoises (Supplementary Fig. S2).

### Stomach content analysis

Stomach content analysis was performed on a total of 103 harbor porpoise stomachs and 61 harbor seal stomachs. Of those, seven harbor porpoise and 20 harbor seal stomachs were empty and excluded from further analysis. Further 24 stomachs were available from gray seals. Unfortunately, all gray seal stomachs were either empty or only contained very few hard parts deemed not representative and therefore no further analysis could be conducted.

#### Harbor porpoise

A total of 7,087 individual prey items, belonging to teleost fish, polychaetes, cephalopods, and crustaceans were identified in all harbor porpoise stomachs (n = 96). Gobies (42.39%) and gadoids (39.58%) had the highest %FO followed by sandeels (38.54%) and flatfish (37.50%) (Supplementary Table S8). In terms of reconstructed biomass, gadoids provided by far the highest proportion (54.73%) followed by flatfish (21.10%) and sandeels (8.57%). All other prey guilds each contributed less than 5% to the consumed biomass. Harbor porpoises gained most of their energy also from gadoids (43.80%), flatfish (22.75%), and sandeels (11.85%) (Supplementary Table S9).

#### Harbor seal

In harbor seals (n = 41), a total of 975 individual prey items, encompassing teleost fish, cephalopods, and crustaceans, were identified. Flatfish (70.73%) exhibited the highest %FO, followed by demersal roundfish (29.27%) and gadoids (26.83%) (Supplementary Table S9). Flatfish also represented the highest proportion of consumed biomass (53.09%), followed by clupeids (17.21%). Harbor seals derived the majority of their energy from flatfish (40.49%) and clupeids (32.05%) (Supplementary Table S10).

#### Energy-rich prey in harbor porpoise

After excluding one outlier with an unusually high biomass of reconstructed high-energy prey (in 2021), model 2, which included gray seal abundance as a predictor, best explained the amount of energy-rich prey (sandeels, pelagic roundfish, and clupeids) in harbor porpoises (n = 86) (Supplementary Table S11). Including gray seal abundances increased the explained deviance from 4.19 to 17.6%. The biomass, which was based solely on high-energy prey, exhibited seasonal, though not significant patterns (GAM; *p* > 0.05), with highest amounts being found in October and lowest in June (Supplementary Fig. S3). We also found that high-energy prey in harbor porpoises was significantly decreasing (GAM; p = 0.01) with increasing gray seal abundance (Supplementary Fig. S3).

### Metabarcoding

A total of 334 stomach and intestine (harbor porpoises) as well as scat (seals) samples were used for metabarcoding analysis, comprising 131 gray seal, 153 harbor seal, and 50 harbor porpoise samples. Among these, one harbor porpoise, two harbor seal, and five gray seal samples exhibited no detectable fish DNA. Following quality filtering (Q > score 20), a cumulative total of 12,026,345 merged sequence reads across all samples were correctly indexed. Of these, 4,639,929 merged sequence reads (38.58%) originated from gray seals, 5,915,909 (49.19%) from harbor seals, and 1,470,507 (12.23%) from harbor porpoises. All negative controls (PCR blanks) showed no DNA presence, thereby affirming the absence of PCR contamination.

Sequence reads were classified into 54 unique OTUs (Supplementary Table S12) belonging to fish species of freshwater, brackish and marine origin. Of those, 88.89% (n = 48) were unambiguously identified to species level with 100% BLAST. Six OTUs were identified as “complex”, meaning they were closely related species either belonging to the same genus (*Alosa* and *Lampetra*) or family (Ammodytidae: greater sandeel (*Hyperoplus lanceolatus*) and small sandeel (*Ammodytes tobianus*), Pleuronectidae: European flounder (*Platichthys flesus*) and European plaice (*Pleuronectes platessa*); long-rough dab (*Hippoglossoides platessoides*) and dab (*Limanda limanda*); Triglidae: grey gurnard (*Eutrigla gurnardus*) and red gurnard (*Chelidonichthys cuculus*)).

The majority of OTUs (individual prey species’ sequences) were found in samples from harbor seal (n = 47), followed by gray seal (n = 40) and harbor porpoise (n = 27) (Supplementary Table S13). Overall prey composition in terms of %FO exposed one sandeel species (lesser sandeel (*Ammodytes marinus*), 45.80%) and two demersal roundfish species (*A. cataphractus* and *C. lyra*, 38.93% and 44.27%, respectively) as most important prey species for gray seals (Supplementary Table S13). The complex OTU of *P. flesus*/*P. platessa* had by far the highest %FO (53.59%) followed by one goby species (sand goby (*Pomatoschistus minutus*), 39.22%) and one demersal roundfish species (shorthorn sculpin (*Myoxocephalus scorpius*), 36.60%) in harbor seals. Harbor porpoises had highest %FO for two goby species (Lozano’s goby (*Pomatoschistus lazanoi*) and sand goby (*Pomatoschistus minutus*), 38.30% and 31.91%, respectively) and the OTU complex *H. lanceolatus/A. marinus* (31.91%). Species of the prey guilds demersal roundfish (*C. lyra*), flatfish (*S. solea*) and, clupeids (herring (*Clupea harengus*)) also showed relative high contributions in terms of %FO with 29.79%, 23.40% and 23.40%, respectively.

**Fig. 4:**
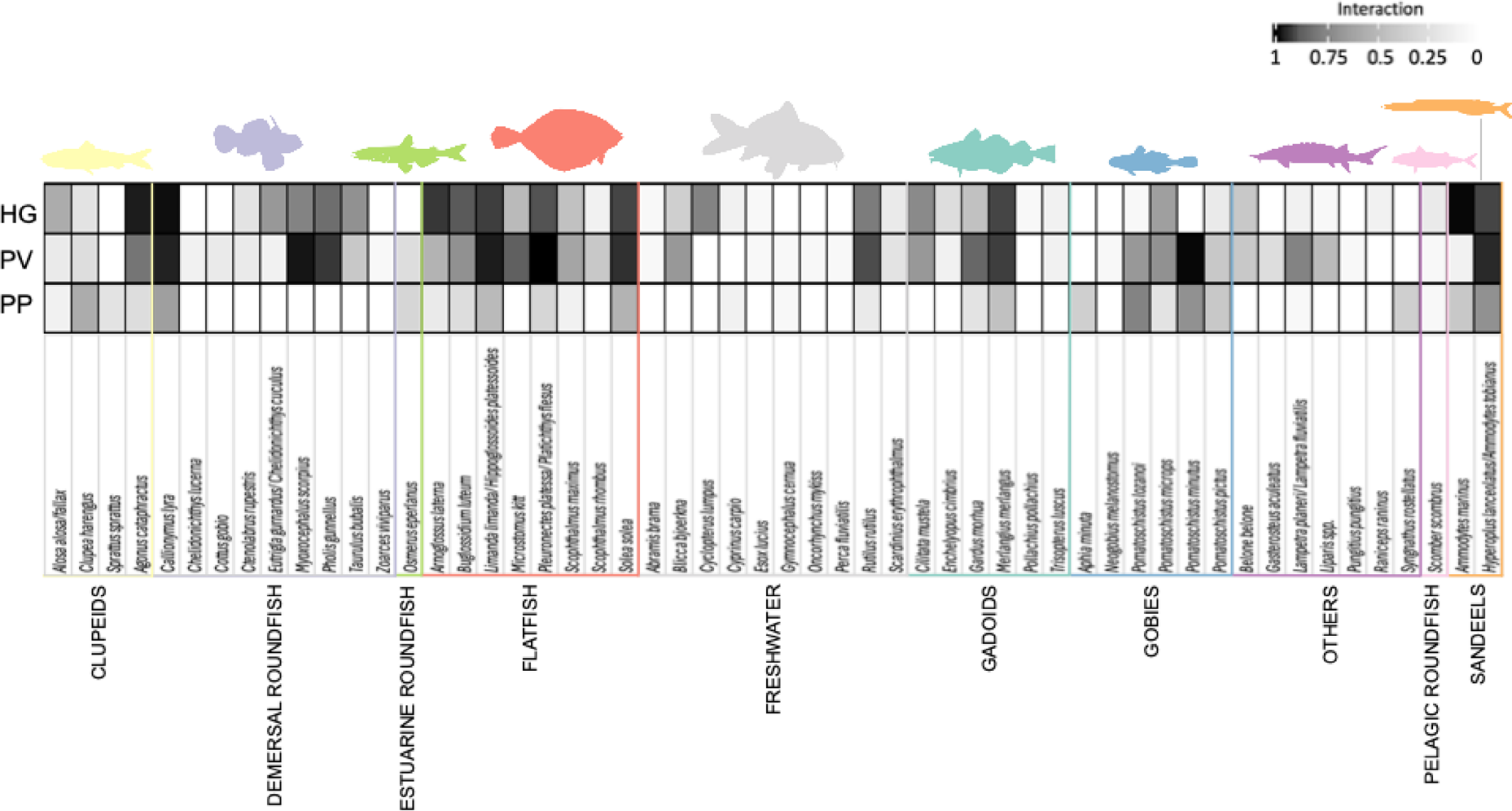
Interaction between the three top predator species (gray seal “HG” [top], harbor seal “PV” [middle] and harbor porpoise “PP” [bottom]) and their prey species (columns) based on results from metabarcoding (% FO)).The shade of each matrix cell is proportional to the number of prey encounters per predator (interaction). Prey guild color-coding is the same as in Fig. 3.

The interaction between top predators and prey species was highest for species belonging to demersal roundfish, flatfish, gobies and sandeels (Fig. 4).

Harbor and gray seal prey taxa appeared very similar (Jaccard similarity index = 0.71). Similarity for harbor porpoise with both seal species appeared to be the same (Jaccard similarity index = 0.46 and 0.45, respectively).

## Discussion

This study examined trophic similarity and resource overlap among gray seals, harbor seals, and harbor porpoises in the southern North Sea. Our findings suggest substantial dietary overlap, especially for energy-rich prey such as sandeels. According to ecological theory, such overlap among species occupying similar trophic levels can intensify interspecific competition, particularly when environmental conditions shift^69^. Gray seals, with their broader trophic niche, may hold a competitive advantage, especially if prey becomes more limited. In recent years, gray seals have been recognized to predate on harbor seals and porpoises, and even conspecifics^25,70,71^, reinforcing their dominant ecological role.

The increase in gray seal abundance over recent decades coincides with a narrowing of the isotopic niches of harbor seals and porpoises, indicating increasing trophic pressure. Disentangling the role of competition in shaping predator population dynamics remains difficult, especially in the absence of fine-scale prey availability data^72^. Nevertheless, overlapping prey use between seals and porpoises supports the inference of competitive interactions. While sandeels remain a common prey item, seals also consume more higher trophic level prey such as flatfish and demersal roundfish, contributing to their higher trophic positions. In contrast, harbor porpoises more frequently target lower-trophic-level prey like clupeids and small sandeels^73^. The main dietary overlap in high-trophic prey likely centers on juvenile whiting (0–20 cm), which are commonly found in the diets of all three predators.

A substantial decline in clupeid biomass in porpoise stomachs, from over 12,000 g to less than 600 g between the two study periods, may partly explain the observed increase in porpoise trophic position. Isotopic data also revealed seasonal variation in prey use (δ^34^S in harbor seals, δ^15^N in gray seals), although interpretation is limited by small sample sizes per season, particularly for gray seals. These patterns likely reflect changes in prey availability or foraging behavior and merit further investigation^24,74^.

Although seasonality was not the dominant factor shaping diet across all species, substantial shifts in δ^15^N and δ^34^S in gray and harbor seals point to temporal dietary changes. For porpoises, seasonal declines in energy-rich prey, though not statistically significant, suggest a potential vulnerability to short-term resource limitations, especially if competitive pressure from gray seals continues to increase.

Interestingly, harbor seals occupied higher trophic positions than gray seals during the early study period (2000–2012), a pattern that reversed later. This may be due to sample bias; the earlier dataset for gray seals was both smaller and skewed towards juveniles and represented their much lower population size. Contrary to^22^, who reported greater niche separation between gray and harbor seals, our data suggest substantial overlap. This is likely due to our larger sample size and the use of muscle tissue, which integrates dietary signals over longer periods than whole blood^75^.

Dietary differences between gray and harbor seals were minimal, and both species showed high and stable trophic positions with a strong benthic diet signal, suggesting convergence in their foraging strategies over time. In contrast, harbor porpoises consistently relied more on pelagic resources than seals, though their estimated α values also indicate a notable benthic contribution, which is in line with ealier studies, e.g.,^27,60^. For harbor seals in the early period (2000–2012), posterior predictive checks revealed a substantial mismatch between model predictions and observed α values; the observed data showed a sharp peak near α = 1, whereas posterior replicates were more diffuse. However, the inference of an almost exclusively benthic diet remains ecologically plausible in the southern North Sea and aligns with the results of stomach content analyses from this and other studies (e.g.,^23^), which show a near absence of pelagic prey in harbor seals during this period (Supplementary Table S9). Interestingly, earlier studies from around Scotland show that, depending on the season, clupeids were the main component of harbor seal diets in the late 1980s^76,77^, whereas their contribution declined sharply about two decades later^25^. This shift could be linked, among other factors, to differences in foraging habitat or to local variation in clupeid availability.

The broader niche of gray seals aligns with their extensive foraging ranges and evidence of individual dietary specialisation^78,79^. Persistently elevated δ^34^S values over 21 years suggest that some individuals forage well beyond the Wadden Sea, likely including transient individuals from the UK^34,80^. Unfortunately, the lack of sulfur isotope data from prey precluded more robust interpretation.

Prey availability and energetic value are central to understanding dietary patterns of predators. Variability in prey energy density, shaped by species, size, season, and environmental conditions, may influence predator foraging decisions and efficiency^23,81^. Size reductions in key prey species like herring, potentially driven, among others, by fisheries and climate change, are expected to reduce available energy to predators^82^. Such reductions are unlikely to be offset by increases in prey abundance and may disproportionately affect species with higher metabolic demands or restricted foraging ranges^83^.

Notably, isotopic mismatch between harbor seals and demersal prey such as hooknose, viviparous blenny and larger gadoids may reflect either restricted foraging access or dietary specialization. In contrast, the inclusion of all 33 prey species, with some more important than others, within gray seal niches underscores their generalist foraging strategy and potential resilience to shifts of their prey.

While fisheries survey data (e.g., ICES IBTS) suggest that several key prey stocks are stable or increasing (e.g., Atlantic cod, sandeel, whiting), herring has declined, and many non-commercial demersal fish remain unsurveyed^84^. Species like gobies, dragonets, smelt, and hooknose are prevalent in predator diets^23,26,60^, yet largely absent from fisheries monitoring due to limited economic interest. This data gap hinders the assessment of competition as well as energy flow at ecologically relevant scales^85,86^. The observed reduction in energy-rich prey in harbor porpoise diets can be associated with the increasing abundance of gray seals, though the decline in herring spawning stock biomass likely also influenced this trend and warrants further investigations. Our multi-method approach, combining stable isotopes, stomach content analysis, and metabarcoding, demonstrates the value of an integrated dietary assessment^13,27^. While stable isotope analysis enabled a detailed assessment of isotopic niche overlap, metabarcoding provided high-resolution insights into prey species composition and dietary overlap. Stomach content analysis, in turn, allowed in-depth evaluation of biomass intake over time, which could be linked to the expansion of the gray seal niche as detected through SIA. However, limitations in sample availability and uneven seasonal coverage, particularly for porpoise stomachs between 2006 and 2011, restricted temporal resolution. Furthermore, posterior predictive checks of the effects of species and covariates on stable isotope values indicated that model performance varied among isotopes. The model reproduced the distribution of δ^15^N closely, whereas for δ^13^C and δ^34^S it captured the central tendency but did not fully reproduce lower-value tails and secondary structure. This likely reflects underlying ecological heterogeneity not explicitly represented in the model. Results were consistent with non-parametric analyses (Supplementary Table S6), confirming species differences as the primary source of variation, while effects of age, sex, and season were limited and not consistently supported.

Metabarcoding results reinforce the patterns seen in stable isotopes and stomach content analysis, with seal species showing greater prey overlap and broader taxonomic diversity than harbor porpoises. The high Jaccard similarity index (0.71) between gray and harbor seals and lower values for porpoises (< 0.46) further support a pattern of trophic divergence between porpoises and seals, and convergence between the two seal species.

The future trajectory of trophic interactions remains uncertain. Anthropogenic pressures including climate change and overfishing may increasingly influence and accelerate which prey resources are available to marine top predators. Local declines in harbor seal populations along both sides of the North Atlantic have already been linked to rising gray seal populations^87,88^. Harbor seals are also more susceptible to viral epidemics^89^, compounding their sensitivity to environmental and trophic pressures. Additionally, harbor seals in the southern North Sea may have reached their carrying capacity^90^. Neither the number of stranded harbor seals nor the pathological examinations conducted in Germany and Denmark indicate a disease-related decline in the population^91^. A plausible explanation for this could be that young animals are unable to find sufficient prey and therefore do not reach adulthood, but this would need further investigation. How harbor porpoises may be affected under increased competition remains unclear and should be addressed through future work.

## Conclusion

Although food resources currently appear sufficient for gray seals, harbor seals, and harbor porpoises in the southern North Sea, this balance may shift if primary and secondary production declines, fish stocks continue to change, or fish communities undergo further functional reorganization^13,92^. Given indications that harbor seals might already have reached their carrying capacity, they may be particularly vulnerable to any future reductions in prey availability and increases in competition. Our findings reveal substantial dietary overlap among these predators, particularly for energy-rich prey. The recovery and subsequent growth of the gray seal population appears to compress the trophic niche of harbor seals and porpoises, suggesting increasing competitive pressure.

To better understand the ecological roles of these top predators and their influence on energy transfer within the marine food web, continued monitoring is essential to capture changes in trophic interactions and potential competitive dynamics under ongoing environmental change. Future research should combine dietary data with bioenergetic models. These tools can estimate prey demands, energy budgets, and potential resource limitations under changing environmental conditions and anthropogenic stressors.

This study is the first to assess trophic niche overlap among the three species using an integrated approach that includes stomach content analysis, metabarcoding, and stable isotope analysis. All methods point to partial dietary convergence, particularly in prey selection and foraging habitat. Notably, we have identified a potential link between reduced energy-rich prey availability for harbor porpoises and the increasing abundance of gray seals. These results highlight the importance of multi-method approaches for unraveling predator interactions in dynamic marine ecosystems.

## Supplementary Information

Below is the link to the electronic supplementary material.

Supplementary Material 1

## Data Availability

The raw sequencing data generated in this study have been deposited in the NCBI Sequence Read Archive (SRA) under Bioproject ID PRJNA1423236: https://dataview.ncbi.nlm.nih.gov/object/PRJNA1423236?reviewer=uj63qt79087fuv07nuff6pc23r.
