# Supplementary material for: Rising competition among North Sea mammalian top predators: a multi-method perspective on trophic ecology
Source: Sci Rep. 2026 May 14;16:15172. doi: 10.1038/s41598-026-53094-2 (PMC13176342; doi:10.1038/s41598-026-53094-2)
Supplement: Supplementary file 1 — Supplementary Material 1 [file 41598_2026_53094_MOESM1_ESM.docx]

### Supplementary material

**Rising competition among North Sea mammalian top predators: a multi-method perspective on trophic ecology**

Eileen Heße, Joy Ometere Boyi, Krishna Das, Kristina Lehnert, Mathilde Piette, Marianna Pinzone, Ursula Siebert, Anita Gilles

**Supplementary Table S1** Overview of the number of samples available per species for the three different methods. Numbers in bold give the total number of samples available per method. Note that some individuals had samples available for two or three methods. SIA = stable isotope analysis, SCA = stomach content analysis.

|  | | **Method** | | |
| --- | --- | --- | --- | --- |
| **Species** | | **SIA** | **SCA** | **metabarcoding** |
| Gray seals | *Halichoerus grypus* | 55 | 24 | 165 |
| Harbor seals | *Phoca vitulina* | 133 | 61 | 118 |
| Harbor porpoises | *Phocoena phocoena* | 112 | 103 | 50 |
|  |  | **300** | **189** | **333** |

**Supplementary Table S2** Classification of harbor seal and gray seal age classes based on their length, sex and strandings date.

|  | **pup** | **juvenile** | **adult** |
| --- | --- | --- | --- |
| **Gray seal ♂** | stranded 1. NOV - 31. JAN | up to 174 cm | from 175 cm |
| **Gray seal ♀** | stranded 1. NOV - 31. JAN | until 158 cm | from 156 cm |
| **Harbor seal ♂** | stranded 1. JUN - 31. AUG | until 142 cm | from 143 cm |
| **Harbor seal ♀** | stranded 1. JUN - 31. AUG | until 129 cm | from 130 cm |

**Supplementary Table S3** Stable Isotope Analysis - Mean isotope values of nitrogen (δ^15^N), carbon (δ^13^C; corrected for the Suess effect to the year 2020), and sulphur (δ^34^S) per species, age class and sex. Values for period 1 (2000-2012) are shown on top and values for period 2 (2013-2021) on the bottom.

| **Species** | **Age** | **Sex** | **2000-2012** | | | | | |
| --- | --- | --- | --- | --- | --- | --- | --- | --- |
|  |  |  | **δ^15^N** | **SD** | **δ^13^C.cor** | **SD** | **δ^34^S** | **SD** |
| **Gray seal** | adult | ♀ | 16.5 | 0.0 | -18.4 |  | 15.1 |  |
|  | adult | ♂ | 18.3 | 0.9 | -18.5 | 1.6 | 17.7 | 1.5 |
|  | juvenile | ♀ | 18.3 | 1.9 | -17.5 | 0.7 | 17.7 | 3.0 |
|  | juvenile | ♂ | 18.6 | 1.9 | -17.4 | 0.7 | 16.3 | 2.4 |
| **Harbor porpoise** | adult | ♀ | 16.6 | 1.4 | -18.9 | 1.6 | 13.3 | 1.6 |
|  | adult | ♂ | 15.0 | 2.8 | -18.6 | 2.0 | 15.0 | 1.4 |
|  | juvenile | ♀ | 16.8 | 1.3 | -19.0 | 1.3 | 15.0 | 2.5 |
|  | juvenile | ♂ | 17.3 | 1.7 | -19.0 | 2.1 | 14.3 | 2.6 |
| **Harbor seal** | adult | ♀ | 19.6 | 0.8 | -16.2 | 1.1 | 15.9 | 1.7 |
|  | adult | ♂ | 18.5 | 2.6 | -16.6 | 1.6 | 16.3 | 1.9 |
|  | juvenile | ♀ | 19.4 | 1.3 | -16.9 | 2.1 | 14.6 | 1.9 |
|  | juvenile | ♂ | 19.2 | 1.5 | -19.6 | 5.9 | 14.2 | 1.7 |
|  |  |  | **2013-2021** | | | | | |
|  |  |  | **δ^15^N** | **sd** | **δ^13^C.cor** | **sd** | **δ^34^S** | **sd** |
| **Gray seal** | adult | ♀ | 18.2 | 1.2 | -18.7 | 3.9 | 17.9 | 5.9 |
|  | adult | ♂ | 17.5 | 1.4 | -17.9 | 1.2 | 20.6 | 1.0 |
|  | juvenile | ♀ | 19.8 | 1.4 | -17.4 | 0.7 | 20.4 | 3.4 |
|  | juvenile | ♂ | 19.1 | 1.5 | -17.5 | 0.7 | 19.6 | 2.0 |
| **Harbor porpoise** | adult | ♀ | 17.7 | 0.8 | -17.5 | 0.5 | 18.8 | 1.0 |
|  | adult | ♂ | 17.6 | 0.6 | -17.7 | 0.6 | 19.2 | 1.2 |
|  | juvenile | ♀ | 17.5 | 1.3 | -18.0 | 0.9 | 19.1 | 1.7 |
|  | juvenile | ♂ | 16.9 | 1.8 | -18.2 | 0.8 | 19.4 | 1.7 |
| **Harbor seal** | adult | ♀ | 18.9 | 1.1 | -16.5 | 0.8 | 17.7 | 1.1 |
|  | adult | ♂ | 18.2 | 1.3 | -17.6 | 1.9 | 17.5 | 2.4 |
|  | juvenile | ♀ | 18.8 | 0.9 | -16.8 | 0.5 | 18.0 | 0.7 |
|  | juvenile | ♂ | 18.7 | 0.9 | -16.9 | 0.7 | 18.2 | 0.8 |

**Supplementary Table S4** Stable Isotope Analysis - Mean isotopic values for carbon (δ^13^C) and nitrogen (δ^15^N) of all available fish prey species sampled between 2019 and 2021 in the FishNet project ^1^. Whiting and Atlantic cod are divided into two size classes (<10cm and >10cm). Where possible species were divided into species feed on pelagic (p) species or benthic (b) species. Species highlighted in grey have not been included in Heße et al. (2025). Prey codes correspond to abbreviations used in Figure 3 of the main manuscript.

| **Species** | **Code** | **Prey guild** | **δ^13^C mean** | **δ^15^N mean** | **δ^13^C SD** | **δ^15^N SD** | **n** |
| --- | --- | --- | --- | --- | --- | --- | --- |
| **Herring (p)** | CH | Clupeids | -18.9 | 13.7 | 1.0 | 2.0 | 12 |
| **European pilchard** | SP | Clupeids | -18.7 | 14.6 | 0.4 | 1.0 | 4 |
| **Sprat (p)** | Sspr | Clupeids | -18.9 | 15.3 | 0.7 | 1.2 | 15 |
| **Twaite spp.** | Aspp | Clupeids | -18.9 | 15.3 | 1.8 | 1.2 | 3 |
| **Common dragonet (p)** | CL | Demersal roundfish | -19.3 | 16.2 | 0.8 | 1.3 | 14 |
| **Grey gurnard** | EG | Demersal roundfish | -17.4 | 17.6 | 0.5 | 1.4 | 13 |
| **Hooknose** | AG | Demersal roundfish | -15.9 | 17.4 | 1.0 | 1.2 | 96 |
| **Viviparous blenny** | ZV | Demersal roundfish | -15.5 | 18.1 | 1.1 | 0.9 | 13 |
| **Smelt** | OE | Estuarine roundfish | -17.8 | 17.2 | 1.3 | 1.2 | 26 |
| **Solenette** | BL | Flatfish | -16.6 | 16.5 | 1.0 | 0.7 | 34 |
| **Common dab (p)** | LL | Flatfish | -19.3 | 15.3 | 0.2 | 2.3 | 3 |
| **Flounder** | PF | Flatfish | -16.1 | 16.8 | 1.7 | 1.1 | 3 |
| **Plaice** | PP | Flatfish | -16.3 | 15.8 | 1.1 | 1.4 | 125 |
| **Common sole** | Ssol | Flatfish | -16.5 | 17.0 | 1.1 | 0.7 | 10 |
| **Mediterranean scaldfish** | AL | Flatfish | -16.6 | 16.5 | 0.8 | 0.8 | 40 |
| **Lemon sole** | MK | Flatfish | -17.7 | 15.7 | 0.9 | 1.0 | 18 |
| **Atlantic cod (<10cm)** | GM(<10cm) | Gadoids | -19.0 | 15.6 | 1.0 | 0.6 | 11 |
| **Atlantic cod (>10cm)** | GM(>10cm) | Gadoids | -16.0 | 18.3 | 0.3 | 0.7 | 5 |
| **Whiting (<10cm)** | MM(<10cm) | Gadoids | -18.1 | 15.8 | 1.0 | 1.5 | 56 |
| **Whiting (>10cm)** | MM(>10cm) | Gadoids | -16.0 | 17.9 | 0.8 | 0.9 | 57 |
| **Norway pout** | TE | Gadoids | -18.8 | 12.9 | 0.5 | 1.1 | 12 |
| **Fourbeard rockling** | EC | Gadoids | -16.7 | 17.5 | 0.6 | 0.3 | 11 |
| **Sand goby** | PM | Gobies | -17.1 | 17.5 | 0.6 | 0.7 | 58 |
| **Painted goby** | Ppic | Gobies | -17.0 | 17.3 | 0.4 | 0.3 | 9 |
| **Brown shrimp 1** | CCoff | Invertebrates | -17.3 | 16.2 | 2.2 | 0.7 | 6 |
| **Brown shrimp 2** | CCin | Invertebrates | 16.1 | 16.1 | 1.2 | 1.3 | 9 |
| **Seasnail spp.** | Lspp | Others | -16.9 | 16.0 | 0.5 | 0.9 | 24 |
| **Butterfish** | PG(b) | Others | -16.5 | 17.5 | 0.7 | 1.0 | 21 |
| **Butterfish (p)** | PG(p) | Others | -18.5 | 15.9 | 0.4 | 0.4 | 5 |
| **Nilson's pipefish** | SR | Others | -17.1 | 16.2 | 0.4 | 1.0 | 16 |
| **Atlantic mackerel** | Ssco | Pelagic roundfish | -18.5 | 16.1 | 0.9 | 1.0 | 18 |
| **Lesser sandeel (b)** | AM(b) | Sandeels | -17.1 | 17.0 | 1.4 | 0.6 | 4 |
| **Lesser sandeel (p)** | AM(p) | Sandeels | -19.1 | 15.5 | 0.6 | 1.1 | 7 |
| **Greater sandeel (p)** | HL | Sandeels | -19.0 | 15.7 | 0.8 | 1.3 | 10 |

**Supplementary Table S5** Stable Isotope Analysis - Effects of species, age, sex, and season on stable isotope values (δ^13^C, δ^15^N, δ^34^S). Estimates are posterior means with 95% credible intervals. Reference levels: species = gray seal, sex = female, age = juvenile, season = winter. Species abbreviations are pp = harbor porpoise, pv = harbor seal and hg = gray seal.

## δ^13^C

| **Predictor** | **Estimate** | **95% CI** |
| --- | --- | --- |
| Intercept | -17.62 | [-19.59, -15.44] |
| Species (pp) | -0.4 | [-0.90, 0.12] |
| Species (pv) | 0.61 | [0.14, 1.09] |
| Sex (male) | -0.3 | [-0.66, 0.04] |
| Age (adult) | 0.37 | [0.01, 0.75] |
| Season (autumn) | 0.47 | [-0.12, 1.06] |
| Season (spring) | -0.02 | [-0.47, 0.42] |
| Season (summer) | -0.46 | [-0.96, 0.05] |

## δ^15^N

| **Predictor** | **Estimate** | **95% CI** |
| --- | --- | --- |
| Intercept | 19.28 | [17.73, 20.83] |
| Species (pp) | -1.41 | [-1.87, -0.93] |
| Species (pv) | -0.01 | [-0.45, 0.42] |
| Sex (male) | -0.42 | [-0.74, -0.11] |
| Age (adult) | -0.07 | [-0.39, 0.26] |
| Season (autumn) | -0.17 | [-0.70, 0.36] |
| Season (spring) | -0.53 | [-0.92, -0.14] |
| Season (summer) | -0.18 | [-0.63, 0.25] |

## δ^34^S

| **Predictor** | **Estimate** | **95% CI** |
| --- | --- | --- |
| Intercept | 18.38 | [15.21, 21.83] |
| Species (pp) | -1.0 | [-1.61, -0.39] |
| Species (pv) | -1.72 | [-2.30, -1.14] |
| Sex (male) | 0.34 | [-0.08, 0.75] |
| Age (adult) | 0.11 | [-0.32, 0.54] |
| Season (autumn) | -0.04 | [-0.74, 0.67] |
| Season (spring) | 0.35 | [-0.17, 0.88] |
| Season (summer) | -0.04 | [-0.63, 0.55] |

## Residual correlations

| **Predictor** | **Estimate** | **95% CI** |
| --- | --- | --- |
| δ^13^C – δ^15^N | 0.17 | [0.06, 0.28] |
| δ^13^C – δ^34^S | 0.16 | [0.05, 0.27] |
| δ^15^N – δ^34^S | -0.24 | [-0.34, -0.13] |

## Random effects (time period)

| **Predictor** | **Estimate** | **95% CI** |
| --- | --- | --- |
| SD (δ^13^C intercept) | 1.22 | [0.11, 4.43] |
| SD (δ^15^N intercept) | 0.77 | [0.01, 3.77] |
| SD (δ^34^S intercept) | 2.75 | [0.95, 6.98] |

**Supplementary Table S6** Stable Isotope Analysis - Kruskal-Wallace test results per predator species for all three isotopes (δ^13^C, δ^15^N, and δ^34^S). Variables tested were age class, sex, season, and study period.

| **Porpoises** | **Age** | | | **Sex** | | | **Season** | | | **Period** | | |
| --- | --- | --- | --- | --- | --- | --- | --- | --- | --- | --- | --- | --- |
|  | **chi-square** | **df** | **p** | **chi-square** | **df** | **p** | **chi-square** | **df** | **p** | **chi-square** | **df** | **p** |
| δ^15^N | 3.6637 | 1 | 0.05561 | 0.37053 | 1 | 0.5427 | 7.4698 | 3 | 0.05834 | 9.2619 | 2 | **<0.01** |
| δ^13^C | 13.641 | 1 | **<0.01** | 1.4201 | 1 | 0.2334 | 0.79055 | 3 | 0.8517 | 6.4104 | 2 | **<0.01** |
| δ^34^S | 0.01457 | 1 | 0.9039 | 1.8057 | 1 | 0.179 | 2.2316 | 3 | 0.5258 | 32.833 | 2 | **<0.01** |
|  |  |  |  |  |  |  |  |  |  |  |  |  |
| **Harbour seals** | **Age** | | | **Sex** | | | **Season** | | | **Period** | | |
|  | **chi-square** | **df** | **p** | **chi-square** | **df** | **p** | **chi-square** | **df** | **p** | **chi-square** | **df** | **p** |
| δ^15^N | 0.079115 | 1 | 0.7785 | 0.4822 | 1 | 0.4874 | 8.0465 | 3 | 0.08989 | 24.379 | 2 | **<0.01** |
| δ^13^C | 5.9495 | 1 | **0.01472** | 3.2066 | 1 | 0.07334 | 7.905 | 3 | 0.09512 | 6.5225 | 2 | 0.08878 |
| δ^34^S | 2.1429 | 1 | 0.1432 | 2.0627 | 1 | 0.1509 | 19.004 | 3 | **<0.01** | 62.407 | 2 | **<0.01** |
|  |  |  |  |  |  |  |  |  |  |  |  |  |
| **Grey seals** | **Age** | | | **Sex** | | | **Season** | | | **Period** | | |
|  | **chi-square** | **df** | **p** | **chi-square** | **df** | **p** | **chi-square** | **df** | **p** | **chi-square** | **df** | **p** |
| δ^15^N | 7.9611 | 1 | **<0.01** | 1.0249 | 1 | 0.3114 | 20.081 | 3 | **<0.01** | 1.7715 | 2 | 0.4124 |
| δ^13^C | 0.25894 | 1 | 0.6108 | 0.85736 | 1 | 0.3545 | 3.8444 | 3 | 0.2788 | 3.4441 | 2 | 0.1787 |
| δ^34^S | 0.18905 | 1 | 0.6637 | 0.027027 | 1 | 0.8694 | 5.4682 | 3 | 0.1406 | 12.416 | 2 | **<0.01** |

**Supplementary Table S7** Stable Isotope Analysis - Results of convex hulls (TA), standard ellipse areas (SEA) and corrected standard ellipse areas (SEAc) for isotope combinations of δ^13^C/δ^15^N, δ^34^S/δ^15^N and δ^13^C/δ^34^S per predator species. Results are given per study period (2000-2012 versus 2013-2021).

|  |  | **2000 – 2012** | | | **2013 - 2021** | | |
| --- | --- | --- | --- | --- | --- | --- | --- |
|  |  | **Harbour porpoises** | **Harbour seals** | **Grey seals** | **Harbour porpoises** | **Harbour seals** | **Grey seals** |
| **δ^13^C/ δ^15^N** |  |  |  |  |  |  |  |
|  | **TA** | 18.2 | 36.7 | 10 | 24.9 | 22.6 | 22.4 |
|  | **SEA** | 5.8 | 7.5 | 4.7 | 3 | 2.7 | 5.9 |
|  | **SEAc** | 6.2 | 7.7 | 5.19 | 3 | 2.7 | 6.1 |
| **δ^34^S/ δ^15^N** |  |  |  |  |  |  |  |
|  | **TA** | 20 | 33.3 | 16.2 | 37 | 26.3 | 53 |
|  | **SEA** | 7.9 | 9.3 | 8.9 | 5.4 | 3.2 | 12.9 |
|  | **SEAc** | 8.5 | 9.6 | 9.8 | 5.5 | 3.3 | 13.3 |
| **δ^13^C/ δ^34^S** |  |  |  |  |  |  |  |
|  | **TA** | 22.6 | 34.1 | 13.1 | 30 | 23.4 | 53.3 |
|  | **SEA** | 9.1 | 10.7 | 7 | 3.4 | 2.9 | 9.4 |
|  | **SEAc** | 9.7 | 11 | 7.7 | 3.5 | 2.9 | 9.6 |

**Supplementary Table S8** Stable Isotope Analysis - Overlap and non-overlap of the maximum likelihood fitted standard ellipses (40%) and 95% ellipses of PP (harbor porpoise), PV (harbor seal) and HG (gray seal) for the two study periods (2000 – 2012 versus 2013 - 2021).

|  | **2000 - 2012** | | | | **2013 - 2021** | | | |
| --- | --- | --- | --- | --- | --- | --- | --- | --- |
|  | **area 1** | **area 2** | **overlap** | **non-overlapping area** | **area 1** | **area 2** | **overlap** | **non-overlapping area** |
| **d^13^C/d^15^N 40%** |  |  |  |  |  |  |  |  |
| PP vers. PV | 6.18 | 7.71 | 0.82 | 0.06 | 3.05 | 2.68 | 0.31 | 0.06 |
| PP vers. HG | 6.18 | 5.19 | 2.13 | 0.23 | 3.05 | 6.08 | 1.49 | 0.2 |
| PV vers. HG | 7.71 | 5.19 | 3.59 | 0.39 | 2.68 | 6.07 | 1.87 | 0.27 |
| **d^13^C/d^15^N 95%** |  |  |  |  |  |  |  |  |
| PP vers. PV | 37.05 | 46.17 | 24.59 | 0.42 | 18.25 | 16.08 | 10.18 | 0.42 |
| PP vers. HG | 37.05 | 31.12 | 24.68 | 0.57 | 18.25 | 36.36 | 16.08 | 0.43 |
| PV vers. HG | 46.17 | 31.11 | 27.45 | 0.55 | 16.08 | 36.36 | 16.08 | 0.44 |
| **d^34^S/d^15^N 40%** |  |  |  |  |  |  |  |  |
| PP vers. PV | <0.01 | <0.01 | <0.01 | <0.01 | 5.5 | 3.27 | 1.05 | 0.14 |
| PP vers. HG | <0.01 | <0.01 | <0.01 | <0.01 | 5.5 | 13.24 | 2.48 | 0.15 |
| PV vers. HG | 9.57 | 9.81 | 4.86 | 0.33 | 3.27 | 13.24 | 2.65 | 0.19 |
| **d^34^S/d^15^N 95%** |  |  |  |  |  |  |  |  |
| PP vers. PV | 50.92 | 57.33 | 25.32 | 0.31 | 32.94 | 19.57 | 16.66 | 0.46 |
| PP vers. HG | 50.92 | 58.76 | 24.99 | 0.3 | 32.94 | 79.34 | 30.24 | 0.37 |
| PV vers. HG | 57.33 | 58.76 | 44.92 | 0.63 | 19.57 | 79.34 | 19.57 | 0.25 |
| **d^13^C/d^34^S 40%** |  |  |  |  |  |  |  |  |
| PP vers. PV | 9.67 | 11.02 | 3.65 | 0.21 | 3.45 | 2.91 | 0.31 | 0.05 |
| PP vers. HG | 9.67 | 7.66 | 3.08 | 0.22 | 3.45 | 9.58 | 3.45 | 0.36 |
| PV vers. HG | 11.02 | 7.66 | 3.16 | 0.2 | 2.91 | 9.58 | 0.71 | 0.06 |
| **d^13^C/d^34^S 95%** |  |  |  |  |  |  |  |  |
| PP vers. PV | 57.91 | 66.03 | 42.93 | 0.53 | 20.68 | 17.44 | 11.14 | 0.41 |
| PP vers. HG | 57.91 | 45.92 | 37.33 | 0.56 | 20.68 | 57.41 | 20.68 | 0.36 |
| PV vers. HG | 66.03 | 45.92 | 35.03 | 0.46 | 17.44 | 57.41 | 15.75 | 0.27 |

**Supplementary Table S9** Stomach Content Analysis - Overview of prey composition on species level for stranded harbor porpoises (n = 127). Frequency of occurrence (FO) and percentage frequency of occurrence (%FO) are given for all stomachs and for all prey species. Index of relative importance (IRI) is given for the different prey guilds. Totals are given per prey guild. Prey guilds were based on indexing by Leopold et al. (2015).

| **Species** |  | **Prey guild** | **n stomachs** | **%FO (n) stomachs** | **n prey** | **%FO (n) prey** | **Mass(g)** | **%Mass** | **Energy(kJ)** | **%Energy** | **IRI** |
| --- | --- | --- | --- | --- | --- | --- | --- | --- | --- | --- | --- |
| Herring | *Clupea harengus* | Clupeids | 12 | 12.50 | 114 | 1.61 | 3332.88 | 2.40 | 33995.36 | 5.93 | 50.2 |
| Sprat | *Sardina pilchardus* | Clupeids | 12 | 12.50 | 167 | 2.36 | 1521.65 | 1.10 | 9890.75 | 1.72 | 43.2 |
| Twaite spp. | *Alosa fallax* | Clupeids | 2 | 2.08 | 2 | 0.03 | 368.94 | 0.27 | 4427.31 | 0.77 | 0.6 |
|  |  | **Clupeids** | **21** | **21.88** | **283** | **3.99** | **5223.47** | **3.77** | **48313.41** | **8.42** | **169.8** |
| Dragonet | *Callionymus lyra* | Demersal roundfish | 17 | 17.71 | 108 | 1.52 | 2943.98 | 2.12 | 15308.68 | 2.67 | 64.6 |
| Dragonet spp. | *Callionymus spp.* | Demersal roundfish | 3 | 3.13 | 47 | 0.66 | 669.22 | 0.48 | 3479.92 | 0.61 | 3.6 |
| Hooknose | *Agonus cataphractus* | Demersal roundfish | 1 | 1.04 | 4 | 0.06 | 47.12 | 0.03 | 208.76 | 0.04 | 0.1 |
|  |  | **Demersal roundfish** | **21** | **21.88** | **159** | **2.24** | **3660.32** | **2.64** | **18997.37** | **3.31** | **106.8** |
| Smelt | *Osmerus eperlanus* | Estuarine roundfish | 15 | 15.63 | 548 | 7.73 | 6242.27 | 4.50 | 31024.06 | 5.41 | 191.2 |
|  |  | **Estuarine roundfish** | **15** | **15.63** | **548** | **7.73** | 6242.27 | **4.50** | 31024.06 | **5.41** | **191.2** |
| Common dab | *Limanda limanda* | Flatfish | 16 | 16.67 | 165 | 2.33 | 6251.59 | 4.51 | 18754.77 | 3.27 | 113.9 |
| Common sole | *Solea solea* | Flatfish | 21 | 21.88 | 586 | 8.27 | 20806.38 | 15.01 | 104031.91 | 18.14 | 509.1 |
| Flatfish spp. | *Flatfish spp.* | Flatfish | 6 | 6.25 | 65 | 0.92 | 694.52 | 0.50 | 2083.55 | 0.36 | 8.9 |
| Flounder | *Platichthys flesus* | Flatfish | 2 | 2.08 | 12 | 0.17 | 335.31 | 0.24 | 1005.93 | 0.18 | 0.9 |
| Plaice | *Pleuronectes platessa* | Flatfish | 2 | 2.08 | 16 | 0.23 | 191.09 | 0.14 | 1126.36 | 0.20 | 0.8 |
| Plaice/Flounder | *P. platessa/P. flesus* | Flatfish | 4 | 4.17 | 59 | 0.83 | 791.43 | 0.57 | 2615.92 | 0.46 | 5.8 |
| Solenette | *Buglossidium luteum* | Flatfish | 3 | 3.13 | 17 | 0.24 | 115.22 | 0.08 | 576.10 | 0.10 | 1.0 |
| Witch | *Glyptocephalus cynoglossus* | Flatfish | 2 | 2.08 | 23 | 0.32 | 77.13 | 0.06 | 308.54 | 0.05 | 0.8 |
|  |  | **Flatfish** | **36** | **37.50** | **943** | **13.31** | **29262.67** | **21.10** | **130503.07** | **22.75** | **1290.4** |
| Atlantic cod | *Gadus morhua* | Gadoids | 19 | 19.79 | 284 | 4.01 | 56405.58 | 40.68 | 174293.24 | 30.39 | 884.4 |
| Five-bearded rockling | *Ciliata mustela* | Gadoids | 1 | 1.04 | 8 | 0.11 | 146.49 | 0.11 | 1040.06 | 0.18 | 0.2 |
| Gadoid spp. | *Gadoid spp.* | Gadoids | 3 | 3.13 | 9 | 0.13 | 849.06 | 0.61 | 3708.21 | 0.65 | 2.3 |
| Poor cod | *Trisopterus minutus* | Gadoids | 1 | 1.04 | 2 | 0.03 | 59.80 | 0.04 | 304.97 | 0.05 | 0.1 |
| Whiting | *Merlangius merlangus* | Gadoids | 21 | 21.88 | 444 | 6.26 | 18425.81 | 13.29 | 71860.64 | 12.53 | 427.7 |
|  |  | **Gadoids** | **38** | **39.58** | **747** | **10.54** | **75886.73** | **54.73** | **251207.11** | **43.80** | **2583.6** |
| Black goby | *Gobius niger* | Gobies | 1 | 1.04 | 4 | 0.06 | 2.20 | 0.00 | 8.50 | 0.00 | 0.1 |
| Goby spp. | *Pomatoschistus spp.* | Gobies | 23 | 23.96 | 1320 | 18.63 | 2739.71 | 1.98 | 10575.27 | 1.84 | 493.6 |
| Sand goby | *Pomatoschistus minutus* | Gobies | 21 | 21.88 | 1680 | 23.71 | 2574.09 | 1.86 | 9935.99 | 1.73 | 559.2 |
|  |  | **Gobies** | **42** | **43.75** | **3004** | **42.39** | **5316.00** | **3.83** | **20519.76** | **3.58** | **2022.2** |
| Brown shrimp | *Crangon crangon* | Invertebrates | 10 | 10.42 | 99 | 1.40 | 99.00 | 0.07 | 434.61 | 0.08 | 15.3 |
| Crab spp. | *Crab spp.* | Invertebrates | 2 | 2.08 | 22 | 0.31 | 0.00 | 0.00 | 0.00 | 0.00 | 0.6 |
| Hermit crab | *Pagurus bernhardus* | Invertebrates | 3 | 3.13 | 3 | 0.04 | 0.00 | 0.00 | 0.00 | 0.00 | 0.1 |
|  |  | **Invertebrates** | **15** | **15.63** | **124** | **1.75** | **99.00** | **0.07** | **434.61** | **0.08** | **28.5** |
| Cottidae | *Cottidea spp.* | Others | 1 | 1.04 | 1 | 0.01 | 67.61 | 0.05 | 0.00 | 0.00 | 0.1 |
| European sturgeon | *Acipenser sturio* | Others | 2 | 2.08 | 2 | 0.03 | 122.72 | 0.09 | 462.65 | 0.08 | 0.2 |
| Pipefish spp. | *Syngnathus spp.* | Others | 2 | 2.08 | 10 | 0.14 | 0.00 | 0.00 | 0.00 | 0.00 | 0.3 |
|  |  | **Others** | **5** | **5.21** | **13** | **0.18** | **190.33** | **0.14** | **462.65** | **0.08** | **1.7** |
| Atlantic horse mackerel | *Trachurus trachurus* | Pelagic roundfish | 1 | 1.04 | 6 | 0.08 | 823.50 | 0.59 | 3862.20 | 0.67 | 0.7 |
|  |  | **Pelagic roundfish** | **1** | **1.04** | **6** | **0.08** | 823.50 | **0.59** | 3862.20 | **0.67** | **0.7** |
| Nereis longissimi | *Nereis longissimi* | Polychaetes | 4 | 4.17 | 25 | 0.35 | 55.12 | 0.04 | 242.53 | 0.04 | 1.6 |
| Nereis spp. | *Nereis spp.* | Polychaetes | 1 | 1.04 | 1 | 0.01 | 0.00 | 0.00 | 0.00 | 0.00 | 0.0 |
|  |  | **Polychaetes** | **5** | **5.21** | **26** | **0.37** | **55.12** | **0.04** | **242.53** | **0.04** | **2.1** |
| Greater sandeel | *Hyperoplus lanceolatus* | Sandeels | 2 | 2.08 | 177 | 2.50 | 1891.54 | 1.36 | 10819.60 | 1.89 | 8.0 |
| Sandeel spp. | *Ammodytes spp.* | Sandeels | 34 | 35.42 | 1000 | 10.65 | 9234.19 | 1.68 | 52819.55 | 2.39 | 436.7 |
| Small sandeel | *Ammodytes marinus* | Sandeels | 1 | 1.04 | 52 | 0.73 | 758.56 | 0.55 | 4338.95 | 0.76 | 1.3 |
|  |  | **Sandeels** | **37** | **38.54** | **1229** | **17.34** | **11884.28** | **8.57** | **67978.10** | **11.85** | 998.7 |
| European common squid | *Loligo vulgaris* | Squid | 2 | 2.08 | 4 | 0.06 | 11.77 | 0.01 | 45.89 | 0.01 | 0.1 |
| Sepiola spp. | *Sepiola spp.* | Squid | 1 | 1.04 | 1 | 0.01 | 0.10 | 0.00 | 0.48 | 0.00 | 0.0 |
|  |  | **Squid** | **3** | **3.13** | **5** | **0.07** | **11.87** | **0.01** | **46.37** | **0.01** | **0.2** |
|  |  |  |  |  |  |  |  |  |  |  |  |
| **TOTAL** |  |  | **96** |  | **7087** | **100** | **138655.55** | **100** | **573591.25** | **100** |  |

**Supplementary Table S10** Stomach Content Analysis - Overview of prey composition on species level for harbor seals (n = 41). Frequency of occurrence (FO) and percentage frequency of occurrence (%FO) are given for all stomachs and for all prey species*.* Index of relative importance (IRI) is given for the different prey guilds. Totals are given per prey guild. Prey guilds were based on indexing by Leopold et al. (2015).

| **Species** |  | **Prey guild** | **n stomachs** | **%FO stomachs** | **n prey** | **%FO prey** | **Mass(g)** | **%Mass** | **Energy(kJ)** | **%Energy** | **IRI** |
| --- | --- | --- | --- | --- | --- | --- | --- | --- | --- | --- | --- |
| Herring | *Clupea harengus* | Clupeids | 3 | 7.32 | 124 | 12.72 | 6153.04 | 16.51 | 62760.96 | 30.90 | 213.90 |
| Sardine | *Sardina pilchardus* | Clupeids | 1 | 2.44 | 1 | 0.10 | 144.94 | 0.39 | 916.04 | 0.45 | 1.20 |
| Twaite shad | *Alosa fallax* | Clupeids | 1 | 2.44 | 1 | 0.10 | 115.86 | 0.31 | 1426.71 | 0.70 | 1.01 |
|  |  | **Clupeids** | **4** | **9.76** | **126** | **12.92** | **6413.83** | **17.21** | **65103.71** | **32.05** | **294.03** |
| Bull rout | *Myoxocephalus scorpius* | Demersal roundfish | 3 | 7.32 | 32 | 3.28 | 141.65 | 0.38 | 627.51 | 0.31 | 26.80 |
| Dragonet | *Callionymus lyra* | Demersal roundfish | 3 | 7.32 | 4 | 0.41 | 88.80 | 0.24 | 461.75 | 0.23 | 4.75 |
| Dragonet spp. | *Callionymus spp.* | Demersal roundfish | 1 | 2.44 | 4 | 0.41 | 15.26 | 0.04 | 79.33 | 0.04 | 1.10 |
| Hooknose | *Agonus cataphractus* | Demersal roundfish | 2 | 4.88 | 31 | 3.18 | 315.13 | 0.85 | 1396.01 | 0.69 | 19.64 |
| Viviparous blenny | *Zoarces viviparous* | Demersal roundfish | 4 | 9.76 | 10 | 1.03 | 824.54 | 2.21 | 10323.73 | 5.08 | 31.60 |
|  |  | **Demersal roundfish** | **12** | **29.27** | **81** | **8.31** | **1385.37** | **3.72** | **12888.31** | **6.35** | **351.98** |
| Smelt | *Osmerus eperlanus* | Estuarine roundfish | 5 | 12.20 | 113 | 11.59 | 4060.79 | 10.90 | 20182.14 | 9.94 | 274.26 |
|  |  | **Estuarine roundfish** | **5** | **12.20** | **113** | **11.59** | **4060.79** | **10.90** | **20182.14** | **9.94** | **274.26** |
| Brill | *Scophthalmus rhombus* | Flatfish | 2 | 4.88 | 2 | 0.21 | 890.50 | 2.39 | 2992.09 | 1.47 | 12.66 |
| Common dab | *Limanda limanda* | Flatfish | 12 | 29.27 | 89 | 9.13 | 4881.86 | 13.10 | 14645.58 | 7.21 | 650.67 |
| Common sole | *Solea solea* | Flatfish | 10 | 24.39 | 62 | 6.36 | 6777.09 | 18.19 | 43696.01 | 21.51 | 598.75 |
| Flatfish spp. | *Flatfish spp.* | Flatfish | 4 | 9.76 | 10 | 1.03 | 530.55 | 1.42 | 1591.64 | 0.78 | 23.90 |
| Flounder | *Platichthys flesus* | Flatfish | 9 | 21.95 | 26 | 2.67 | 2505.43 | 6.72 | 7516.30 | 3.70 | 206.15 |
| Plaice | *Pleuronectes platessa* | Flatfish | 4 | 9.76 | 17 | 1.74 | 1637.61 | 4.40 | 9498.14 | 4.68 | 59.89 |
| Plaice/Flounder | *P. platessa/P. flesus* | Flatfish | 4 | 9.76 | 9 | 0.92 | 304.08 | 0.82 | 912.25 | 0.45 | 16.97 |
| Turbot/Brill | *S. maximus/rhombus* | Flatfish | 3 | 7.32 | 3 | 0.31 | 2252.86 | 6.05 | 1390.26 | 0.68 | 46.50 |
| Witch | *Glyptocephalus cynoglossus* | Flatfish | 1 | 2.44 | 1 | 0.10 | na | na | na | na | na |
|  |  | **Flatfish** | **29** | **70.73** | **219** | **22.46** | **19779.99** | **53.09** | **82242.29** | **40.49** | **5343.89** |
| Atlantic cod | *Gadus morhua* | Gadoids | 5 | 12.20 | 41 | 4.21 | 1854.87 | 4.98 | 5731.55 | 2.82 | 112.00 |
| Five-bearded rockling | *Ciliata mustela* | Gadoids | 1 | 2.44 | 1 | 0.10 | 17.12 | 0.05 | 68.50 | 0.03 | 0.36 |
| Whiting | *Merlangius merlangus* | Gadoids | 7 | 17.07 | 86 | 8.82 | 2647.11 | 7.10 | 10323.73 | 5.08 | 271.90 |
|  |  | **Gadoids** | **11** | **26.83** | **128** | **13.13** | **4519.10** | **12.13** | **16123.77** | **7.94** | **677.64** |
| Goby spp. | *Pomatoschistus spp.* | Gobies | 4 | 9.76 | 149 | 15.28 | 446.26 | 1.20 | 1722.58 | 0.85 | 160.78 |
| Sand goby | *Pomatoschistus minutus* | Gobies | 3 | 7.32 | 104 | 10.67 | 126.39 | 0.34 | 487.85 | 0.24 | 80.53 |
|  |  | **Gobies** | **6** | **14.63** | **253** | **25.95** | **572.65** | **1.54** | **2210.43** | **1.09** | **402.23** |
| Brown shrimp | *Crangon crangon* | Invertebrates | 7 | 17.07 | 23 | 2.36 | 23.00 | 0.06 | 100.97 | 0.05 | 41.33 |
| Crab spp. | *Crab spp.* | Invertebrates | 1 | 2.44 | 2 | 0.21 | na | na | na | na | na |
| Hermit crab | *Pagurus bernhardus* | Invertebrates | 1 | 2.44 | 2 | 0.21 | na | na | na | na | na |
|  |  | **Invertebrates** | **9** | **21.95** | **27** | **2.77** | **23.00** | **0.06** | **100.97** | **0.05** | **62.14** |
| Garfish | *Belone belone* | Others | 1 | 2.44 | 3 | 0.31 | na | na | na | na | na |
| Lesser pipefish | *Syngnathus rostellatus* | Others | 1 | 2.44 | 2 | 0.21 | 0.48 | 0.00 | na | na | na |
| Pipefish spp. | *Syngnathus spp.* | Others | 1 | 2.44 | 1 | 0.10 | 0.24 | 0.00 | na | na | na |
| Unknown | *unknown* | Others | 4 | 9.76 | 13 | 1.33 | 315.87 | 0.85 | 3705.08 | 1.82 | 21.28 |
|  |  | **Others** | **5** | **12.20** | **19** | **1.95** | **316.59** | **0.85** | **3705.08** | **1.82** | **34.13** |
| Greater sandeel | *Hyperoplus lanceolatus* | Sandeels | 1 | 2.44 | 2 | 0.21 | 44.56 | 0.12 | 258.45 | 0.13 | 0.79 |
| Lesser sandeel | *Ammodytes tobianus* | Sandeels | 2 | 4.88 | 4 | 0.41 | 49.21 | 0.13 | 285.43 | 0.14 | 2.65 |
| Small sandeel | *Ammodytes marinus* | Sandeels | 1 | 2.44 | 1 | 0.10 | 2.10 | 0.01 | 12.16 | 0.01 | 0.26 |
|  |  | **Sandeels** | **2** | **4.88** | **7** | **0.72** | **95.87** | **0.26** | **556.04** | **0.27** | **4.76** |
| Long finned squid | *Loligo forbesii* | Squid | 1 | 2.44 | 2 | 0.21 | 90.22 | 0.24 | na | na | 1.09 |
|  |  | **Squid** | **1** | **2.44** | **2** | **0.21** | **90.22** | **0.24** | **na** | **na** | **1.09** |
|  |  |  |  |  |  |  |  |  |  |  |  |
| **TOTAL** |  |  | **41** |  | **975** | **100.00** | **37257.42** | **100.00** | **203112.74** | **100.00** |  |

**Supplementary Table S11** Stomach Content Analysis - Model output for GAMs explaining the amount of high-energy prey guilds (clupeids, sandeels and pelagic roundfish) in the diet of harbor porpoises (n = 86).

| **Absolute prey mass high-energy prey guilds: Model 1 (deviance explained 4.19%)** | | | | | | | |
| --- | --- | --- | --- | --- | --- | --- | --- |
| APMHEPG ~ s (sex, bs = "re") + s(age, bs = "re") + s(month, k = 5, bs = "cc") ) | | | | | | | |
|  | **Estimate** | **SE** | **t value** | **p-value** |  |  |  |
| **Intercept** | 5.2273 | 0.38 | 13.87 | **<2e-16** |  |  |  |
|  |  |  |  |  |  |  |  |
|  | **edf** | **Ref.df** | **F** | **p-value** |  |  |  |
| **s(sex)** | <0.01 | 1 | 0 | 0.557 |  |  |  |
| **s(age)** | 0.494 | 1 | 0.516 | 0.298 |  |  |  |
| **s(month)** | 0.838 | 3 | 0.224 | 0.477 |  |  |  |
| **Absolute prey mass high-energy prey guilds: Model 2 (deviance explained 17.6%)** | | | | | | | |
| APMHEPG ~ s (sex, bs = "re") + s(age, bs = "re") + s(month, k = 5, bs = "cc") + s((sqrt(hg_abundance)),  k = 4, bs = "cs") | | | | | | | |
|  | **Estimate** | **SE** | **t value** | **p-value** |  |  |  |
| **Intercept** | 5.0598 | 0.3334 | 15.17 | **<2e-16** |  |  |  |
|  |  |  |  |  |  |  |  |
|  | **edf** | **Ref.df** | **F** | **p-value** |  |  |  |
| **s(sex)** | 0.001 | 1 | 0.000 | 0.5533 |  |  |  |
| **s(age)** | 0.421 | 1 | 0.452 | 0.2910 |  |  |  |
| **s(month)** | 1.511 | 3 | 0.711 | 0.2726 |  |  |  |
| **s(hg_abundance)** | 1.094 | 2 | 3.149 | **0.0112** |  |  |  |

**Supplementary Table S12** Metabarcoding - Overview of all detected sequences per operational taxonomic unit (OTU) applying the 16S rRNA fish primer (n = 54). Rows with two species names indicate a “complex” OTU, meaning that sequences belong to closely related species, either to the same genus

| **Species/OTU** | **OTU sequence** |
| --- | --- |
| Abramis_brama | TAAGGTACAAAATTTAACCACGTTAAACGACTCCATAGAAAGCAAGAACTTAATGGTGAATAAAATTTTACC |
| Agonus cataphractus | TTAGACGTCTAAGCAGCCTACGTTAAGCACCCTGAATAAAGGACTAAACAAAGTGAACCCTGCTATAATGTC |
| Alosa alosa/Alosa fallax | TTAGACGCCAACCAACCACGAAAAGCGGCCGCTGACTGGACCCTTAAACAACGTGATTATGGCATAAGCGTC |
| Ammodytes marinus | TTAGACGCCAAGACAGACCATGTTAAACACCTCCCCACAAGGAGCCCAAACCAAATGGTCCCTGCCCTAGTGTC |
| Aphia minuta | TAAGACACTAGGTTAGCCTACGTTAAAAATCCTCGGATATTAGGAGTAAACCAAGTAGCCTCTATCCTCCTGTC |
| Arnoglossus laterna | TTAGACCCCGGAGTAGCTCATGTTAATAGACCTTGCCCAAGGAAACAACTAAATGAGCCCTACCCCTGTGTC |
| Belone belone | TTAGACAAAAGACAGACCATGTTAAACAAACTGACCTAAACAGAATAAACCTATTGGCCTACTGCCTAGATGTC |
| Blicca bjoerkna | TAAGGTACAAATTCAACCACGTTAAACGACTCCATAGAAAGCAAGAACTTAGTGGCGAATGAAATTTTACC |
| Buglossidium luteum | TCAGACGAAAAGACAGTCCGTCCCCACTACCCTGGATAAAGGAACCAGACGGCCCCTGTCTAGATGTC |
| Callionymus lyra | TTAGACAAACCAGGAAAACTTTTAAACCTAAAATAAACAGATTACTTAAGTCTTCACCTCGGAAGTGTC |
| Chelidonichthys_lucerna | TTAGACACTAAGGCAGCTCATGTCAAAAACCCTACAATAAAAGGCTGAACCAAATGAGCTCTGCCCTAATGTC |
| Cilitata mustela | TTAGACCTAAAATAACTCACGTTTAATAACCTGTTTAACCAGCAAAAACTTAGTGATACTTATTGAGGTGTC |
| Clupea harengus | TTAGACGCCCACCAATCACGAAAAGCAGGTCTCGCTCAACAGACTTCCAAACAACGTGATACTGGCACAAACGTC |
| Cottus_gobio | TTAGACACGAAAGCAGCCCACGTTAAGCACCCCGAATAAAGGACTAAACCAAGTGGGCCCTGCCCTAATGTC |
| Ctenolabrus rupestris | TTAGACACCAAAGTAGCCCCTGTTAAACACCCCTGCTTAAAGGACTAAACCGAAGGGGTCCTACCCTAATGTC |
| Cyclopterus lumpus | TTAGACACCAAAGCAGCTTACGTTAAGCACCCCCTAATAAGGGCCTAAACCAAGTAAGCCCTGCCCTACTGTC |
| Cyprinus carpio | TAAGGTACAAAACTCAACCACGTTAAGCAACTCAATAAAAAGCAAAAACCTTGTGGACCATGAGATTTTACC |
| Enchelyopus cimbrius | TTAGACCTAAAGTAATTCACGTTTAATATACTGCACAAACCAGTAAAAACTTAGTGACATTTATTGAGGTGTC |
| Esox_lucius | TTAGACACCCGGCAGACCCTGTTAAATAACTGAACTATCAGATTAAAACAAAGCGGCCCCTGGCCTACATGTC |
| Eutrigla gurnardus/Chelidonichthys cuculus | TTAGACACTAAGGCAGCTCATGTCAAAAACCCTATAATAAAAGACTGAACCAAATGAGCTCTGCCCTAATGTC |
| Gardus morhua | TTAGACCTAAAGTAAGTCACGTTTAACATGCTGTGATAACAGTAAAAACTTAGTGATATTTACTGAAGTGTC |
| Gasterosteus_aculeatus | TTAGACACTAAAGTGGATCATGTCAATGACCCTAAATAAAGGATTGAACAAGATGGAACCCACTCTGATGTC |
| Gymnocephalus cernua | TTAGACACCAAGATAGATCATGTTAAACACCCCTTGTTAAAGGGTTAAACCAAATGAACCCTATCCTACTGTC |
| H. lanceolatus/A. tobianus | TTAGACGCCAAGACAGACCATGTTAAACACCTCCTCATAAGGAGCCCAAACCAAATGGTCCCTGCCCTAGTGTC |
| Lampetra planeri/Lampetra fluviatilis | TCCAAACATTTACATCGCATAATCATTATTCACGATGCACAGTT |
| Limanda limanda/Hippoglossoides platessoides | TTAGACACACAGGTGGCCCATGTCAAATGACCCCCGCTAAGGACCTGAACTAAGTGGAACCTGCCTTGATGTC |
| Liparis spp. | TTAGACACTAAGACAGCTTACGTTAAACACTCCCGAGATAAAAGGAATAAACTAAACAAGCTCTGCCCTAATGTC |
| Merlangius merlangus | TTAGACCTAAGGTAAGTCACGTTTAACATGCTGTGATAACAGAAAAAACTTAGTGATATTTACTGAAGTGTC |
| Microstomus kitt | TTAGACACACAGGTGGACCATGTCAAACACCCCCTACTAAGGGCCTGAACTAAATGGCACCTGCCTTGATGTC |
| Myoxocephalus scorpius | TTAGACACCAAGGTAGCCCACGTTAAAGACCCTGAACAAAGGACTAAACCAAGTGAGCCCTACCCTAATGTC |
| Neogobius_melanostomus | TTAGACACCGAAGCAGACCGCGTTAAGAGCCCCAAACCAAAAGGACTAAACCAAACGAACCCTGCTTTTTTGTC |
| Oncorhynchus_mykiss | TTAGACACCAGGCAGATCACGTCAAGCAACCTTGAATTAACAAGTAAAAACGCAGTGACCCCTAGCCCATATGTC |
| Osmerus eperlanus | TTAGACACTAGACAGCCCACGTTAAATTTCCTCTAAAAAGAGGAAAAAACATTGTGATTCCTGTCTCTCCTGTC |
| Perca_fluviatilis | TTAGACACCAAGACAGATCATGTTAAACACTCCTTAATAAAGGACTAAACCAAATGACCCCTGTCCTAATGTC |
| Pholis gunnellus | TTAGACACCAAGGCAGATCATGTTAATAGTCCTGAATAAAGGAACAAACCAGATGGAATCTGCCCTAATGTC |
| Pleuronectes platessa/Platichthys flesus | TTAGACACACAGGTGGACCATGTCAAATACCCCCAGCTAAGGGCCTGAACTAAATGGAACCTGCCTTGATGTC |
| Pollachius_pollachius | TTAGACCTAAGGTAAGTCACGTTTAATGTGCTATAATAACAGTGAAAACTTAGTGATATTTACTGAAGTGTC |
| Pomatoschistus lozanoi | TAAGACACAAGGCAGCAAACGTTAAAACCCCTGGACCAACAGACACAACCAAGTTAATGCCTGCCCTTTTGTC |
| Pomatoschistus microps | TAAGACACAAGGCAGCGAACGTTAAGACACCTGGACGAACAGCTACAACCAAGTTAGTGCCTGCCCTCTTGTC |
| Pomatoschistus minutus | TAAGACACAAGGCAGCAAACGTTAAGACCCCTGGGTAAACAGACACAACCAAGTTAGTACCTGCCCTTTTGTC |
| Pomatoschistus pictus | TAAGACACAAGGCAGCAAACGTTAAAACCCCTGGGCAAACAGATACAACCAAGTTAATGCCTGCCCTTTTGTC |
| Pungitius_pungitius | TTAGACACTAAAGTGGATCATGTCAATGACCCCTAAACAAAGGATTGAACCAAATGGAACCCACCCTGATGTC |
| Raniceps raninus | TTAGACCTAAAGTAAGCCATGTTTAGTATCCTGAGATATCAGTAAAAACTTAATGGCAATTATTGAAGTGTC |
| Rutilus rutilus | TAAGGTACAAAATTCAGCCACGTTAAATGACTCTACAAAAAGCAAGAACCTAGTGGCCAGTGAAGTTTTACC |
| Scardinius erythrophthalmus | TAAGGTACAAAATTTAACCACGTTAAACGACTCTGTAGAAAGCAAGAACTTAGTGGCGAATGAAGTTTTACC |
| Scomber scombrus | TTAAGACACTAAGCCATATCAAGTTAAACACCCCCAAACAAGGGACTAAACTTATTGAAATCATTGGCCGTATGTC |
| Scophthalmus maximus | TGAGACGATAGGGCAGCCCATGTTAAGTACCCCCCTCACCGGACCAAACTAAATGACCCCTGCTCTAATGTC |
| Scophthalmus rhombus | TGAGACGATAGGGCAGCCCATGTTAAACACCCTTATTAACGGACTAAACTAAATGGCCCCTGCTTTAGTGTC |
| Solea solea | TTAGACAACAATTCAGCCCGTCCCCAAATTCTAAATAAACGAACACACTTAACGGCCCCTGTATTAATGTC |
| Sprattus sprattus | TTAGACGCCCACCAATCATGAAAAGCAAGTCTCAGTTAACAGACCTCCAAACAACATGATACTGGTATAAACGTC |
| Syngnathus rostellatus | TAAGACAATAGGAGGACTTTATGTAGTAAACCTAAAAAGGGCATAAACAAGTTCACACTCCCCCCAATGTC |
| Taurulus bubalis | TTAGACACTAAGGCAGCCCACGTTAAAGACCCTAAATAAAGGATTAAACCAAGCGGGCCCTGCCCTGATGTC |
| Trisopterus luscus | TTAGACCTAAAGTAAATCACGTTTAACATCCTGCAATAATAGTAAAAACTTACTGATACTTACTGAAGTGTC |
| Zoarces viviparous | TTAGACACCAAGACAGATCATGTTAATAACCCTAAATAAAGGCTAAACCAAGTGGAACCTGCCCTAATGTC |

**Supplementary Table S13** Metabarcoding - Frequency of occurrence (FO) and percentage frequency of occurrence (%FO) of prey species, sorted by prey guilds, found in metabarcoding samples of HG (gray seal, n = 165 scats), PV (harbor seal, n = 118 scats) and PP (harbor porpoise, n = 50 stomach and intestinal samples).

|  |  | **HG** | | **PV** | | **PP** | |
| --- | --- | --- | --- | --- | --- | --- | --- |
| **Prey Species** | **Guild** | **FO** | **FO%** | **FO** | **FO%** | **FO** | **FO%** |
| *Alosa alosa/fallax* | Clupeids | 12 | 9.16 | 3 | 1.96 | 2 | 4.26 |
| *Clupea harengus* | Clupeids | 4 | 3.05 | 5 | 3.27 | 11 | 23.40 |
| *Sprattus sprattus* | Clupeids | 0 | 0.00 | 0 | 0.00 | 3 | 6.38 |
| *Agonus cataphractus* | Demersal roundfish | 51 | 38.93 | 23 | 15.03 | 5 | 10.64 |
| *Callionymus lyra* | Demersal roundfish | 58 | 44.27 | 49 | 32.03 | 14 | 29.79 |
| *Chelidonichthys lucerna* | Demersal roundfish | 0 | 0.00 | 2 | 1.31 | 0 | 0.00 |
| *Cottus gobio* | Demersal roundfish | 0 | 0.00 | 3 | 1.96 | 0 | 0.00 |
| *Ctenolabrus rupestris* | Demersal roundfish | 4 | 3.05 | 3 | 1.96 | 0 | 0.00 |
| *Eutrigla gurnardus/Chelidonichthys cuculus* | Demersal roundfish | 15 | 11.45 | 2 | 1.31 | 0 | 0.00 |
| *Myoxocephalus scorpius* | Demersal roundfish | 19 | 14.50 | 56 | 36.60 | 0 | 0.00 |
| *Pholis gunnellus* | Demersal roundfish | 24 | 18.32 | 39 | 25.49 | 0 | 0.00 |
| *Taurulus bubalis* | Demersal roundfish | 16 | 12.21 | 8 | 5.23 | 0 | 0.00 |
| *Zoarces viviparus* | Demersal roundfish | 0 | 0.00 | 1 | 0.65 | 0 | 0.00 |
| *Osmerus eperlanus* | Estuarine roundfish | 0 | 0.00 | 5 | 3.27 | 6 | 12.77 |
| *Arnoglossus laterna* | Flatfish | 39 | 29.77 | 11 | 7.19 | 3 | 6.38 |
| *Buglossidium luteum* | Flatfish | 29 | 22.14 | 16 | 10.46 | 5 | 10.64 |
| *Limanda limanda/ Hippoglossoides platessoides* | Flatfish | 35 | 26.72 | 51 | 33.33 | 10 | 21.28 |
| *Microstomus kitt* | Flatfish | 10 | 7.63 | 28 | 18.30 | 0 | 0.00 |
| *Pleuronectes platessa/Platichthys flesus* | Flatfish | 31 | 23.66 | 82 | 53.59 | 6 | 12.77 |
| *Scophthalmus maximus* | Flatfish | 9 | 6.87 | 13 | 8.50 | 1 | 2.13 |
| *Scophthalmus rhombus* | Flatfish | 2 | 1.53 | 8 | 5.23 | 0 | 0.00 |
| *Solea solea* | Flatfish | 33 | 25.19 | 42 | 27.45 | 11 | 23.40 |
| *Abramis brama* | Freshwater | 1 | 0.76 | 1 | 0.65 | 0 | 0.00 |
| *Blicca bjoerkna* | Freshwater | 7 | 5.34 | 15 | 9.80 | 0 | 0.00 |
| *Cyclopterus lumpus* | Freshwater | 19 | 14.50 | 0 | 0.00 | 0 | 0.00 |
| *Cyprinus carpio* | Freshwater | 2 | 1.53 | 0 | 0.00 | 2 | 4.26 |
| *Esox lucius* | Freshwater | 1 | 0.76 | 1 | 0.65 | 0 | 0.00 |
| *Gymnocephalus cernua* | Freshwater | 0 | 0.00 | 1 | 0.65 | 1 | 2.13 |
| *Oncorhynchus mykiss* | Freshwater | 0 | 0.00 | 1 | 0.65 | 0 | 0.00 |
| *Perca fluviatilis* | Freshwater | 0 | 0.00 | 1 | 0.65 | 0 | 0.00 |
| *Rutilus rutilus* | Freshwater | 22 | 16.79 | 32 | 20.92 | 3 | 6.38 |
| *Scardinius erythrophthalmus* | Freshwater | 3 | 2.29 | 4 | 2.61 | 0 | 0.00 |
| *Cilitata mustela* | Gadoids | 17 | 12.98 | 15 | 9.80 | 0 | 0.00 |
| *Enchelyopus cimbrius* | Gadoids | 5 | 3.82 | 2 | 1.31 | 0 | 0.00 |
| *Gardus morhua* | Gadoids | 9 | 6.87 | 26 | 16.99 | 4 | 8.51 |
| *Merlangius merlangus* | Gadoids | 33 | 25.19 | 35 | 22.88 | 9 | 19.15 |
| *Pollachius pollachius* | Gadoids | 1 | 0.76 | 0 | 0.00 | 0 | 0.00 |
| *Trisopterus luscus* | Gadoids | 2 | 1.53 | 2 | 1.31 | 0 | 0.00 |
| *Aphia minuta* | Gobies | 0 | 0.00 | 0 | 0.00 | 6 | 12.77 |
| *Neogobius melanostomus* | Gobies | 0 | 0.00 | 1 | 0.65 | 0 | 0.00 |
| *Pomatoschistus lozanoi* | Gobies | 1 | 0.76 | 15 | 9.80 | 18 | 38.30 |
| *Pomatoschistus microps* | Gobies | 14 | 10.69 | 16 | 10.46 | 4 | 8.51 |
| *Pomatoschistus minutus* | Gobies | 0 | 0.00 | 60 | 39.22 | 15 | 31.91 |
| *Pomatoschistus pictus* | Gobies | 3 | 2.29 | 8 | 5.23 | 8 | 17.02 |
| *Belone belone* | Others | 8 | 6.11 | 8 | 5.23 | 0 | 0.00 |
| *Gasterosteus aculeatus* | Others | 0 | 0.00 | 5 | 3.27 | 0 | 0.00 |
| *Lampetra planeri/Lampetra fluviatilis* | Others | 2 | 1.53 | 19 | 12.42 | 1 | 2.13 |
| *Liparis spp.* | Others | 1 | 0.76 | 10 | 6.54 | 0 | 0.00 |
| *Pungitius pungitius* | Others | 0 | 0.00 | 1 | 0.65 | 0 | 0.00 |
| *Raniceps raninus* | Others | 2 | 1.53 | 0 | 0.00 | 0 | 0.00 |
| *Syngnathus rostellatus* | Others | 0 | 0.00 | 0 | 0.00 | 6 | 12.77 |
| *Scomber scombrus* | Pelagic roundfish | 3 | 2.29 | 1 | 0.65 | 1 | 2.13 |
| *Ammodytes marinus* | Sandeels | 60 | 45.80 | 2 | 1.31 | 7 | 14.89 |
| *Hyperoplus lanceolatus/Ammodytes tobianus* | Sandeels | 33 | 25.19 | 47 | 30.72 | 15 | 31.91 |

**Supplementary Figure S1** Trophic positions of gray seals (HG), harbor porpoises (PP) and harbor seals (PV) in period 1 (2000 – 2012) and period 2 (2013 – 2021).

**Supplementary Figure S2** Ranges of probability of intra-specific isotopic niche overlap of adult and juvenile gray seals (left), adult and juvenile harbor seals (middle) and adult and juvenile harbor porpoises (right). Confidence intervals are given at 95%. See Figure 3 in the main manuscript for indexing of prey species.


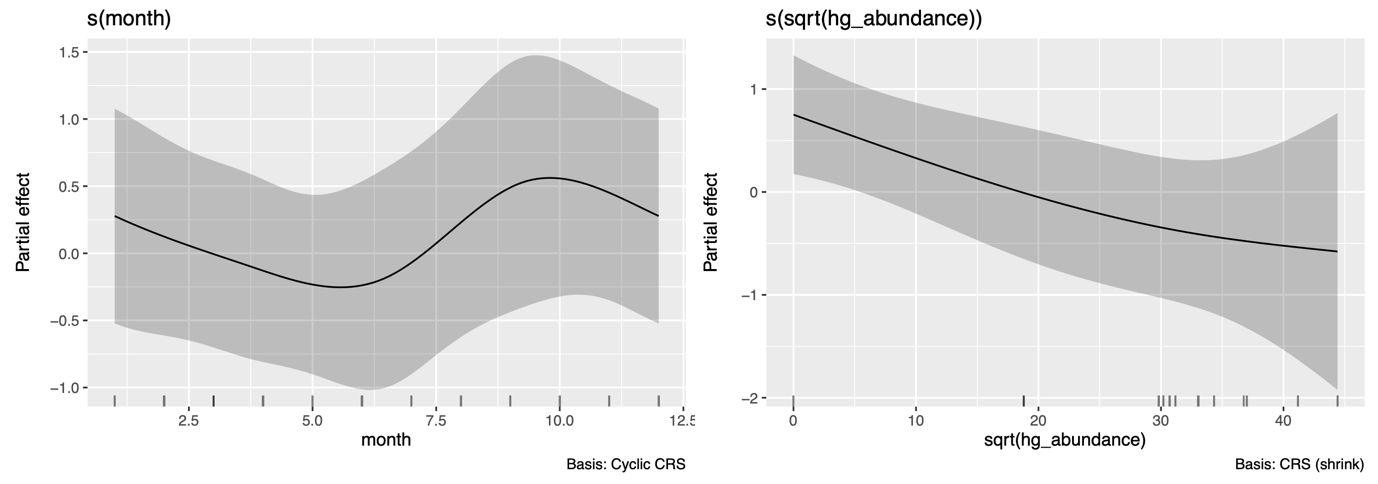


**Supplementary Figure S3** Partial plots of smooth components of the fitted GAM on reconstructed high-energy prey mass (Model 2: n = 86, deviance explained = 17.6%)

References

1 Schückel, S., Bachmann, F., Bruggaier, E., Voigt, C. & Milano, S. Nahrungsnetzprojekt in den Küstengewässern Schleswig-Holsteins mit Fokus auf Fischen (FishNet). Untersuchung und Auswertung zum Mageninhalt und Analyse stabiler Isotope von Benthos- und Fischarten im Rahmen des FishNet Projektes (AP2). 70 (2023).
